# Tuberculosis disease burden in China: a spatio-temporal clustering and prediction study

**DOI:** 10.3389/fpubh.2024.1436515

**Published:** 2025-01-07

**Authors:** Jingzhe Guo, Ce Liu, Fang Liu, Erkai Zhou, Runxue Ma, Ling Zhang, Bin Luo

**Affiliations:** ^1^Institute of Occupational Health and Environmental Health, School of Public Health, Lanzhou University, Lanzhou, China; ^2^Gansu Provincial Center for Disease Prevention and Control, Lanzhou, China

**Keywords:** pulmonary tuberculosis, disease burden, spatial–temporal cluster analysis, prediction study, China

## Abstract

**Introduction:**

The primary aim of this study is to investigate and predict the prevalence and determinants of tuberculosis disease burden in China. Leveraging high-quality data sources and employing a methodologically rigorous approach, the study endeavors to enhance our understanding of tuberculosis control efforts across different regions of China. First, through nationwide spatio-temporal cluster analysis, we summarized the status of tuberculosis burden in various regions of China and explore the differences, thereby providing a basis for formulating more targeted tuberculosis prevention and control policies in different regions; Subsequently, using a time series-based forecasting model, we conducted the first-ever national tuberculosis burden trend forecast to offer scientific guidance for timely adjustments in planning and resource allocation. This research seeks to contribute significantly to China’s existing tuberculosis prevention and control system.

**Materials and methods:**

This research draws upon publicly available pulmonary tuberculosis (PTB) incidence and mortality statistics from 31 provinces and municipalities of mainland China between 2004 and 2018. We organized and classified these data according to province, month, year, and patient age group. Overall, the sample included 14,816,329 new instances of PTB and 42,465 PTB-related fatalities. We used spatiotemporal cluster analysis to record the epidemiological characteristics and incidence patterns of PTB during this period. Additionally, a time series model was constructed to forecast and analyze the incidence and mortality trends of PTB in China.

**Results:**

This study reveals significant regional variations in PTB incidence and mortality in China. Tibet (124.24%) and Xinjiang (114.72%) in western China exhibited the largest percentage change in tuberculosis (TB) incidence, while Zhejiang Province (−50.45%) and Jiangsu Province (−51.33%) in eastern China showed the largest decreases. Regions with significant percentage increases in PTB mortality rates (>100%) included four western regions, six central regions, and five eastern regions. The regions with relatively large percentage decreases in the mortality rate of PTB include Tianjin (−52.25%) and Shanghai (−68.30%). These differences are attributed to two main factors: (1) economic imbalances leading to poor TB control in underdeveloped areas, and (2) differences in TB-related policies among provinces causing uneven distribution of disease risks. Consequently, China may still face challenges in achieving the World Health Organization’s 2030 tuberculosis control goals. Nationwide, the mortality rate of PTB in China increased between 2004 and 2018 (percentage change: 105.35%, AAPC: 4.1), while the incidence of PTB showed a downward trend (percentage change: -20.59%, AAPC: −2.1). Among different age groups, the 0–19 age group has the smallest disease burden. While incidence and mortality from TB were primarily found in adults 60 years of age or older, the age group of 0–19 years has the smallest burden of TB, highlighting obvious differences in age characteristics. It is predicted that the mortality rate of TB in China will continue to increase. In summary, the TB epidemic in China has been largely controlled due to the implementation of many public health programs and policies targeting specific groups and geographical areas. Finding and supporting effective health programs will make it possible to achieve the World Health Organization’s goal of controlling tuberculosis in China.

## 1 Introduction

Tuberculosis is an airborne contagious disease caused by the bacterium *Mycobacterium tuberculosis*. In recent years, TB has become the second deadliest contagious disease globally, surpassed only by COVID-19 (1). According to statistics, since 2019, the global new incidence rate of TB and the number of deaths due to TB have been rising, reversing the long-standing downward trend. In 2022, there were over 10 million new TB patients worldwide, and approximately 1.3 million people died from this disease. TB patients in eight countries account for more than two-thirds of the global total, including India, Indonesia, China, the Philippines, Pakistan and other countries (2). Consequently, TB remains one of the leading causes of adverse health effects and mortality worldwide, particularly in low- and middle-income developing countries.

As a developing country, China has prioritized TB prevention and control in alignment with the recommendations of the World Health Organization (3, 4). China has developed and implemented TB prevention and control programs such as the international widespread adoption of the directly observed treatment, short-course (DOTS) method across different provinces (5), etc. Despite these endeavors, China persists one of the high-burden countries for TB (1), encountering ongoing challenges in TB prevention and control, especially for the main type of pulmonary tuberculosis (PTB), accounting for about 78.7% of tuberculosis cases (6). Therefore, this research focuses on PTB specifically to analyze the Tuberculosis disease burden in China. Current research predominantly delves into the analysis of natural factors, including meteorological factors, seasonal patterns, and geographic locations, and their impact on TB transmission (7–9). Moreover, regional disparities in TB burden are also linked to social factors, including variations in TB-related prevention and control policies across different regions. Therefore, there is an urgent need for nationwide spatiotemporal cluster analysis to provide a basis for comparing the effectiveness of different policies in different regions, thereby pinpointing more targeted TB prevention and control measures. Such an approach will furnish scientific evidence to bolster the efficiency of TB prevention and control efforts, optimizing existing strategies.

Furthermore, predictive studies are pivotal in anticipating future trends in TB burden. Although geospatial predictive models have become increasingly utilized for this purpose (10), time series-based predictive models remain underexplored in research in this realm. In mainland China, leveraging time-series prediction models rooted in historical data, researchers can forecast whether the forthcoming TB burden will align with the World Health Organization’s end-tuberculosis milestones based on the current situation, offering scientific guidance for timely adjustments in planning and resource allocation.

In order to identify the factors causing disparities in TB prevention and control policies in different regions and to furnish theoretical underpinnings for the development of more efficacious and judicious policies and measures for TB prevention, this study aims to achieve several key objectives. Firstly, it seeks to delineate the current landscape of TB burden in China and examine variations in TB burden across different provinces. Additionally, it endeavors to forecast the future TB burden in China and evaluate the nation’s progress toward meeting the targets outlined in the World Health Organization’s End TB Strategy by 2030. To accomplish these aims, a time series forecast model is employed to project future trends in TB burden and provide scientific insights to facilitate timely adjustments in planning and resource allocation.

## 2 Materials and methods

### 2.1 Research data

In particular, PTB remains the predominant form of TB, constituting approximately 85% of all TB cases (1). Therefore, we conduct this study using PTB surveillance data. TB surveillance data on PTB pathogenicity testing and PTB disease burden for 31 provinces and cities in mainland China spanning from 2004 to 2018 were collected from the National Population and Health Scientific Data Sharing Platform - Public Health Scientific Data Center.^1^ This comprehensive dataset encompasses 14,816,329 TB incidence cases and 42,465 TB deaths for analysis. By categorizing annual TB burden data across different age groups, we derived specific TB-related statistics for adolescents (0–19 years), young adults (20–39 years), middle-aged adults (40–59 years), and older adults (≥60 years).

### 2.2 Statistical methods

The statistical methods used in this study encompass three distinct components which are the linear regression, cluster analysis, and projection prediction, respectively. The first part was to assess the burden of TB disease and discerning trends in the change in mainland China as well as across individual provinces. To accomplish this, we utilized the join-point regression model, a robust statistical tool for assessing trends in disease burdens over time (11). Join-point regression does not require the data to be strictly smooth and automatically detects trend change points in the data, which makes it suitable for public health data with non-linear trends. Additionally, the join-point regression model can automatically detect change points in data trends, providing more precise statistical analysis crucial for interpreting and predicting time trends in health-related events (12). We used annual percentage changes (APCs) (Equations 1-1, 1-2), and average annual percentage changes (AAPCs) (Equation 2-1) to assess trends in the morbidity and mortality of TB between 2004 and 2018. APCs were used to calculate segment-specific linear trends throughout the studied year. AAPCs were used to estimate overall changes throughout the entire duration of the study. These calculations were performed using the join-point model according to the following formula (13):

1. APCs

(1-1) $$\ln\left(\mathrm{morbidity}\;\mathrm{or}\;\mathrm{mortality}\right)=\alpha +\beta x+\varepsilon$$

(1-2) $$\mathrm{APCs}=100\times \left(\exp\left(\beta \right)-1\right)$$

1. AAPCs

(2-1) $$\mathrm{AAPCs}=\left\{\exp\left(\sum w_{i}\beta_{i}/\sum w_{i}\right)-1\right\}\times 100$$

Where *x* represents the calendar year. α denotes the constant of the linear fitting; β is expressed as the slope coefficient of each year; ε is the residual error between the estimated and actual values (Equation 1-1). $w_{i}$ is the number of PTB data for each year (Equation 2-1). To assess whether the fluctuation trend in different segments was statistically significant, we compared the AAPC to 0. If the lower limit of the 95% confidence interval (CI) of the AAPC exceeded zero, it indicated an increasing trend in morbidity or mortality. Conversely, if the upper limit of the 95% CI of the AAPC fell below zero, it suggested a decreasing trend in morbidity or mortality.

In the second part, we performed a systematic cluster analysis based on class averaging with Dynamic Time Wraping (DTW) distance (Equations 3-1–3-3) on the time series of incidence and mortality in 31 provinces and cities in China. This analysis aimed to elucidate the diverse patterns of TB incidence and mortality control across different provinces. While Euclidean distance and DTW distance are commonly used matrix distance calculation methods in clustering algorithms (14), Euclidean distance is notably sensitive to small variations in the time axis. Even minor deviations in time series data may result in substantial differences in calculated distances, rendering it less suitable for time series data with varying time lengths. Moreover, the requirement for equal time series lengths presents a significant limitation, necessitating padding or truncation of the time length, which may lead to inaccurate clustering and an inability to effectively handle non-linear changes (15). In contrast, DTW distance mitigates the shortcomings of Euclidean distance by accommodating time shifts and distortions. It aligns sequences by stretching or compressing them along the time axis, thereby offering flexibility to handle temporal offsets and enabling more precise measurements of similarities between time series. Unlike Euclidean distance, DTW distance does not mandate that time series be of the same length, nor does it necessitate preprocessing steps such as padding or truncation of the time axis. Moreover, DTW distance can effectively manage nonlinear distortions through multiple points (16). Given the significant disparities in morbidity and mortality rates among different provinces and time periods according to the PTB surveillance data, DTW distance was selected for utilization in the clustering process, which proceeds as outlined below:

1. DTW

(3-1) $$Q=q_{1},q_{2},q_{3},\ldots ,q_{i},\ldots ,{q_{m}}^{\prime }$$

(3-2) $$C=c_{1},c_{2},c_{3},\ldots ,c_{j},\ldots ,{c_{n}}^{\prime }$$

(3-3) DTWQC=min∑k=1KωkK

1. Systematic clustering by class averaging

(4-1) $$D_{PQ}=\frac{1}{n_{p}n_{q}}\underset{x_{i}\in G_{p}}{\sum }\underset{x_{j}\in G_{q}}{\sum }d_{ij}^{2}$$

In the DTW distance calculation formula, (Equations 3-1–3-3) *Q* and *C* represent any two time series with respective lengths *m* and *n*, $\omega_{k}$ is the distance $DTW\left(q_{i},c_{j}\right)$ between the corresponding points $\left(q_{i},c_{j}\right)$ of the two time series, K is the length of the longer time series. Following the computation of the DTW distance for each time series, we employ the class averaging method to systematically cluster each time series into distinct classes based on the calculated distances. Subsequently, we compute the distance between the new class and other classes using the same method. This iterative process continues as we merge the two closest classes into a new class according to the calculated distance. These steps are repeated until the number of classes approaches 1. At each stage, clustering diagrams are generated to aid in determining the optimal number of classes. In the systematic clustering model (Equation 4-1), the distance between classes is defined as the average of the squared distances between two elements within each class. Assuming that the clustering proceeds to a certain step where $G_{p}$ and $G_{q}$ are merged into a single entity. $D_{PQ}$ represents the distance between $G_{p}$ and $G_{q}$ . Additionally, $n_{p}$ and $n_{q}$ are the number of elements contained within classes $G_{p}$ and $G_{q}$ respectively, while $d_{ij}$ denotes the distance between two elements in $G_{p}$ and $G_{q}$ (Equation 4-1).

In the third part, we applied a projection model to forecast future TB incidence and mortality rates in China. Firstly, five prevalent time series prediction models, ARIMA, PROPHET, GLMNET, RANDOMFOREST, and PROPHET BOOST were individually used (17–19); subsequently, we evaluated the prediction accuracy for both incidence and mortality rates, and found that the ARIMA model exhibited superior predictive efficiency for TB incidence and mortality time series data in China. In addition, the ARIMA model (Equation 5-1) has the characteristics of being easy to interpret and apply, being able to deal with nonlinear and unstable time series, and also being able to take into account the influence of historical data, with high accuracy in predicting the future, which is suitable for this retrospective study. Consequently, the ARIMA model was selected for subsequent predictions, and its formulation is as follows:


(5-1) $$\mathrm{ARIMA}\left(p,d,q\right)\left(P,D,Q\right)\left[m\right]$$

Where, $\left(p,d,q\right)$ are the non-seasonal parameters in the model and $\left(P,D,Q\right)\left[m\right]$ represent the seasonal parameters in the model. p and P indicate the number of lags of the time series data itself (lags) used in the forecasting model, which is also known as AR/Auto-Regressive term; d and D represent the number of orders of differencing required for the time series data to achieve stability, which is also called as the Integrated term; q and Q represent the number of lags of the forecasting error (lags) used in the forecasting model, additionally referred to as the MA/Moving Average term.

## 3 Results

### 3.1 Overall tuberculosis disease burden and changing trends in mainland China in 2004–2018

**Figure 1:**
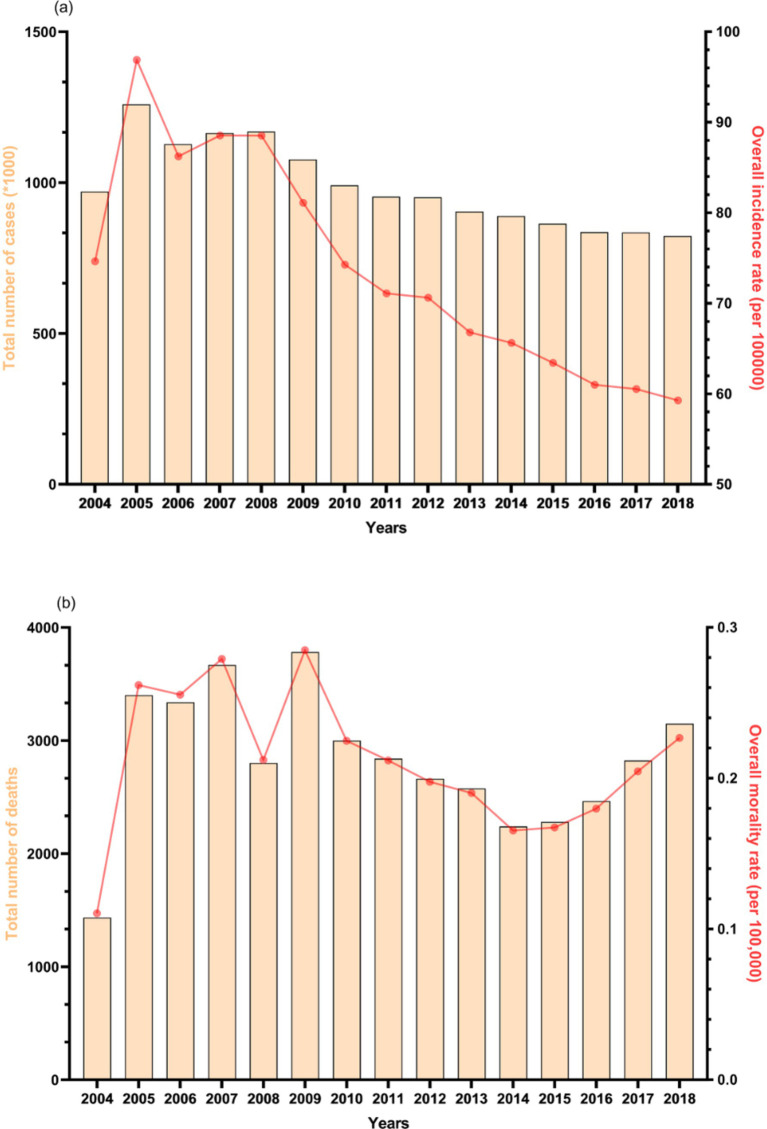
Total situations of **(A)** tuberculosis incidence and **(B)** tuberculosis deaths in mainland China, 2004–2018.

Figure 1 illustrates the yearly fluctuations in new TB cases and TB-related deaths across mainland China from 2004 to 2018. The trend in the number of new cases per year closely mirrors that of the annual incidence rate, both reaching a peak in 2005 at 1,259,308 cases and 96.88 per 100,000 population, respectively, before experiencing a notable decline (Figure 1A). By 2018, the annual incidence rate had reduced to only 61.18% of its 2005 level. Similarly, the trend observed in the number of deaths aligns with that of the annual mortality rate, with both reaching their zenith in 2009 at 3,783 deaths and 0.28 per 100,000 population, respectively. However, following a six-year decline from 2009 to 2014, both indicators began to ascend in 2015 (Figure 1B). Notably, in 2018, the number of deaths and the annual mortality rate stood at 3,149 and 0.23 per 100,000 population, respectively, closely resembling the peak values observed in 2009. In summary, mainland China experienced an overall decrease in incidence percentage change of −20.59% from 2004 to 2018, while witnessing an increasing trend in mortality percentage change, which reached 105.35% (Supplementary Table S1).

**Figure 2:**
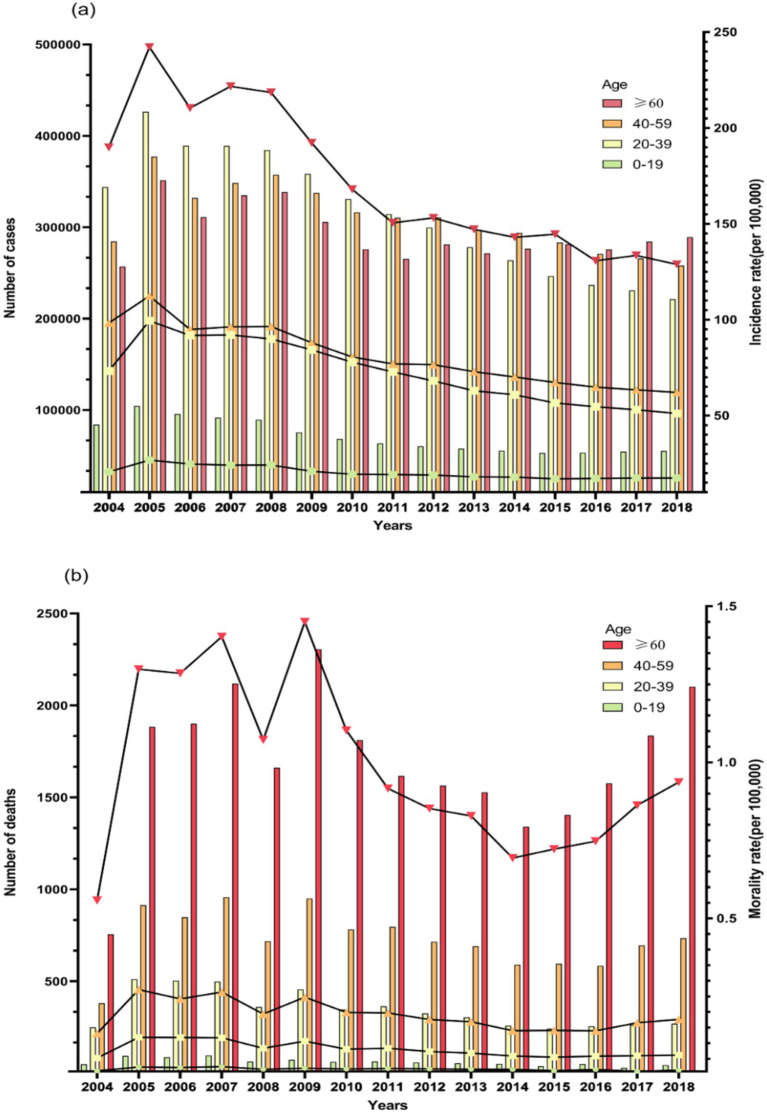
Situations of **(A)** tuberculosis incidence and **(B)** tuberculosis deaths by different age groups in mainland China, 2004–2018.

Figure 2 depicts the distribution of new TB cases and deaths across various age groups in mainland China over the past 15 years, revealing significant age-related disparities. Notably, the burden of TB is lowest in the 0–19 age group, where the annual number of new cases remains below 105,000 and deaths do not exceed 30 per 100,000 annually, both exhibiting a gradual decline. In 2018, the number of new cases in this age group was 54,977, with 44 deaths reported. Between 2004 and 2011, the 20–39 age group exhibited the highest incidence rate among the four age groups, peaking at over 400,000 cases in 2005, though it steadily declined thereafter. Subsequently, the 40–59 age group (from 2002 to 2015) and the age group over 60 years (from 2016 onwards) successively emerged as the primary cohorts for new TB cases, each surpassing 300,000 cases annually. Regarding mortality, the proportionate distribution among the four age groups has remained relatively stable over the years. Specifically, the age group 60 and above consistently exhibits the highest mortality rate, averaging over 1,500 cases per year, while the 40–59 and 20–39 age groups fall within the intermediate range, each averaging around 500 and 300 cases per year, respectively. In summary, the TB burden in mainland China predominantly afflicts individuals aged 60 years and above, markedly surpassing that observed in the 0–19 age group.

### 3.2 Comparison of TB disease burden and changing trends in 31 provinces and cities in mainland China in 2004–2018

**Figure 3:**
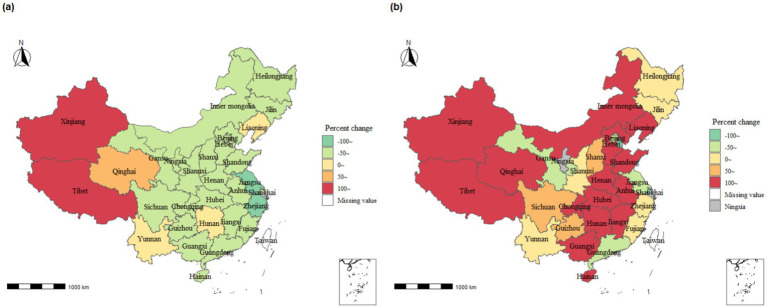
Percentage change in tuberculosis disease burden in mainland China across provinces, 2004–2018. **(A)** Incidence rate; **(B)** Mortality rate.

Figure 3 presents the TB burden distribution among provinces in mainland China, revealing notable geographic disparities in the percentage change of TB burden from 2004 to 2018. Among them, Tibet (124.24%) and Xinjiang (114.72%) exhibited the highest percentage change in TB incidence, displaying a pronounced upward trend, whereas Zhejiang Province (−50.45%) and Jiangsu Province (−51.33%) demonstrated the smallest percentage change in TB incidence, reflecting a prominent downward trend compared to 2004 data (Supplementary Table S1). Significant percentage changes in the mortality rate of pulmonary TB were observed across various regions of mainland China. Notably, the four western regions (Xinjiang 1336.40%, Tibet 364.77%, Qinghai 328.31%, Chongqing 280.20%), six central regions (Anhui 379.22%, Hunan 272.28%, Hubei 196.15%, Henan 124.51%, Inner Mongolia 120.01%, Jiangxi 113.15%), and five eastern regions (Shandong 349.44%, Liaoning 320.85%, Hebei 171.26%, Hainan 157.27%, Guangxi 110.17%) displayed substantial percentage changes in mortality rates. Conversely, Tianjin (−52.25%) and Shanghai (−68.30%) exhibited relatively minor percentage changes in mortality rates. (Notably, due to a lack of mortality data in Ningxia in 2004, its percentage change value was excluded from the statistical calculations, Supplementary Table S1).

From 2004 to 2018, the APCs for TB incidence and mortality in the 31 provinces and cities indicated a statistically significant mean APC and 95% CI of −2.1 (−3.2, −1.0) for incidence (Supplementary Table S2 for specific results), while the AAPC of 4.1 (−1.2, 9.8) was not statistically significant (Supplementary Figures S1, S2). Notably, among the 31 provincial and municipal areas, Jilin, Jiangsu, Zhejiang, and Fujian provinces exhibited significant and statistically significant decreasing trends in morbidity, with AAPCs of −5.1 (−9.1, −0.9), −5.4 (−6.3, −4.6), −5.0 (−6.1, −3.9), and − 5.0, (−9.0, −0.9), respectively (Supplementary Figure S1). Conversely, Xinjiang displayed the most noticeable increasing trend in mortality among the 31 provincial and municipal regions, with an AAPC of 17.7 (7.3, 29) (Supplementary Figure S2).

### 3.3 Time series clustering analysis of the burden of tuberculosis disease in mainland China

The time series analysis of TB disease burden in mainland China was clustered using DTW distance as the clustering index, and the results were presented in Figure 4. The clustering results for TB incidence rates across provinces were categorized into seven major clusters, while the mortality rates were divided into six major clusters (Supplementary Figures S3–S15).

**Figure 4:**
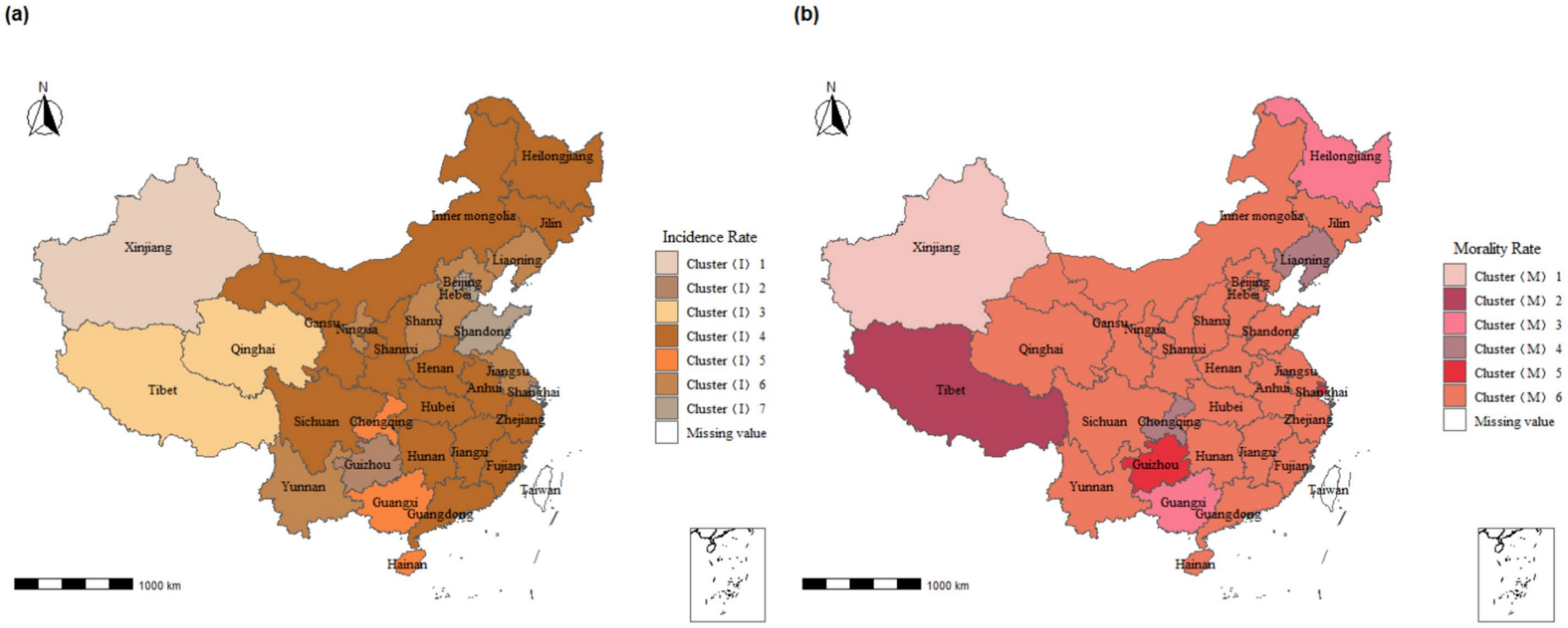
Clustering of tuberculosis incidence and mortality time series analysis mainland China across provinces, 2004–2018. **(A)** Incidence rate; **(B)** Morality rate. *Cluster(I) represents the clustering results of morbidity: (1) fluctuates in 140–310 with a continuous upward trend, (2) fluctuates in 120–190 with a single-peaked change, (3) fluctuates in 70–140 with a continuous upward trend, (4) fluctuates in 50–130 with a continuous downward trend, 5 fluctuates in 140–310 with a continuous upward trend, 6 fluctuates in 70–150 with a continuous downward trend Cluster(M) represents the clustering results of mortality: 1 fluctuates between 0 and 2.5 and fluctuates upwards, 2 fluctuates between 0 and 1.25 and fluctuates downwards with multiple peaks, 3 fluctuates between 0.2–0.7 and fluctuates “U” shaped with multiple peaks, 4 fluctuates between 0.1–0.5 and fluctuates upwards 0.5 and fluctuating upward trend, 5 fluctuating 0.2–0.7 and fluctuating downward trend, 6 fluctuating 0.05–0.5 and multi-peaked fluctuating trend.

Cluster (I) represents the clustering results for morbidity:

- Categories 1, 3, and 5 show a continuous upward trend.
- Category 2 fluctuates between 120 and 190 and exhibits a single-peaked change.
- Categories 4, 6, and 7 show a continuous downward trend.

Cluster (M) represents the clustering results for mortality:

- Categories 1 and 4 exhibit an upward fluctuating trend.
- Categories 2 and 5 exhibit a downward fluctuating trend.
- Category 3 fluctuates between 0.2 and 0.7 and shows a “U” shaped fluctuation with multiple peaks.
- Category 6 fluctuates between 0.05 and 0.5 with multiple peaks, showing no clear upward or downward trend.

The time series analysis of TB incidence rates in most regions of mainland China, primarily the central and eastern regions, falls into Cluster (I)4. The incidence rates in this cluster gradually reduce within a single peak range of 100 to 120 per 100,000 (Supplementary Figure S6). In contrast, the time series analysis of TB mortality in most regions falls into Cluster (M)6, where mortality rates fluctuate within 0–0.5 per 100,000 with multiple peaks (Supplementary Figure S15).

Specifically, we found that the time series clustering of morbidity in Tibet and Qinghai province belongs to Cluster (I)3, while the mortality clustering in these regions belongs to Cluster (M)2 and Cluster (M)6, respectively. The mortality clustering for Guizhou and Shanghai falls into Cluster (M)5, whereas the incidence rate clustering for Guizhou belongs to Cluster (I)2, and Shanghai belongs to Cluster (I)7. Notably, Xinjiang forms a unique cluster, distinct from all other provinces in both morbidity and mortality.

### 3.4 Model fitting and prediction for time series analysis of tuberculosis disease burden in mainland China in 2030

**Figure 5:**
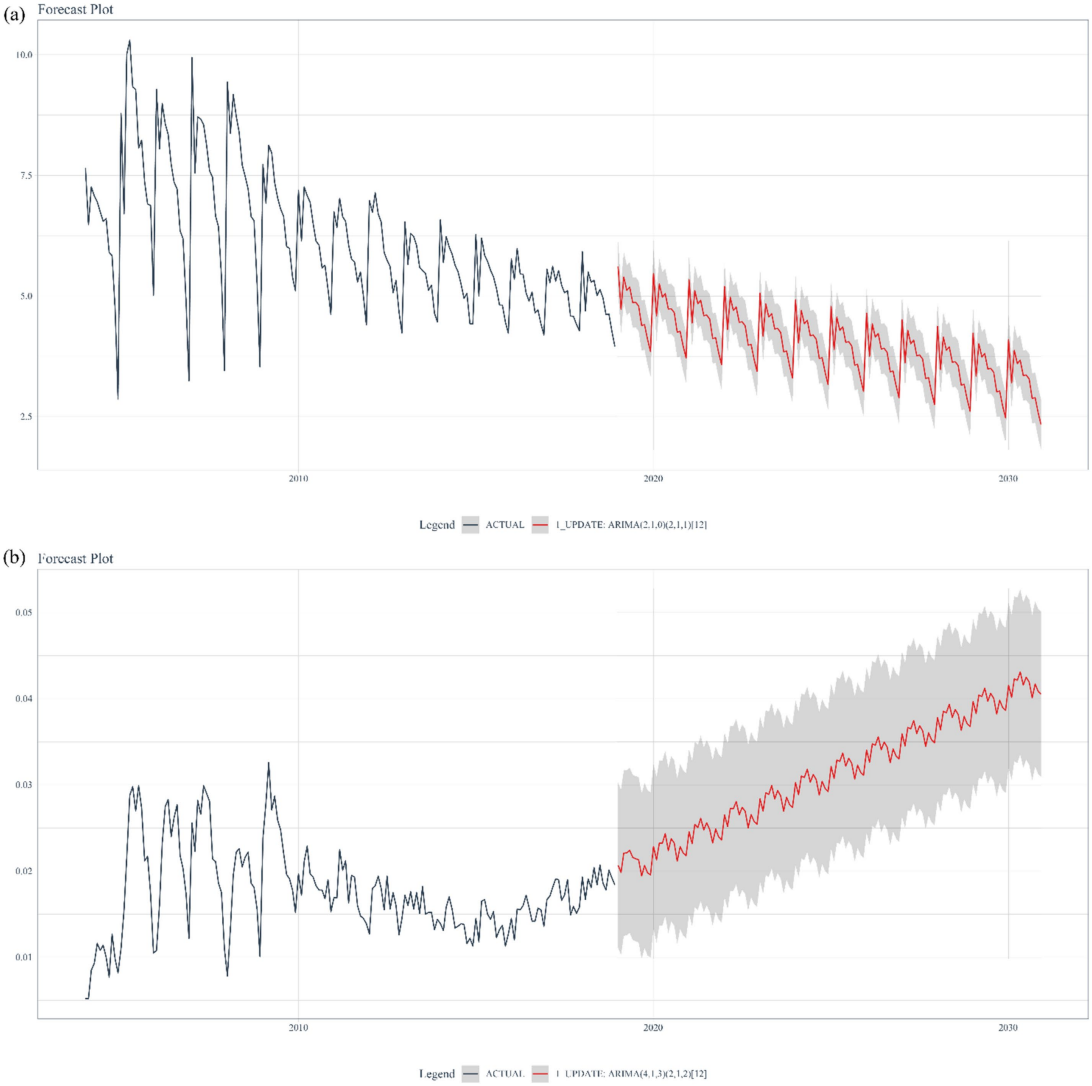
Projection incidence **(A)** and mortality **(B)** of pulmonary tuberculosis in mainland China in 2030.

We found the ARIMA model was the most optimal prediction model for forecasting the TB disease burden in mainland China after testing of the five included models (Supplementary Tables S3, S4). This conclusion was drawn because the ARIMA model demonstrated the best prediction accuracy across several metrics, including MAE, MAPE, MASE, sMAPE, RMSE, and R^2. The final prediction results suggested that the overall incidence rate would continue to decline by 2030, while the mortality rate would continue to increase by 2030 (Figure 5). In addition, the time series analysis of TB disease burden showed an obvious annual cycle and seasonality, with a trough in winter and a peak in spring (Figure 5; Supplementary Figures S16, S17).

## 4 Discussion

In this study, we utilized publicly available data on TB in mainland China provided by the Public Health Science Data Center. We analyzed the spatiotemporal distribution characteristics of TB cases from 2004 to 2018 and conducted the first-ever nationwide predictive research on the burden of TB in China. Regarding tuberculosis burden surveys, previous studies, both domestic and international, primarily focused on descriptive statistical analysis, often limiting their scope to analyzing population prevalence trends. This study goes beyond merely describing changes in disease burden by incorporating cluster analysis based on varying trend patterns. This approach allows for a more nuanced observation of the clustering characteristics of TB burden across regions, facilitating the identification of effective prevention and control strategies. Additionally, the inclusion of predictive analyses enhances the ability to evaluate the effectiveness of existing TB health policies and forecast future trends. The findings of this study indicate that the TB prevention and control policies in Guizhou Province and Xinjiang are particularly effective and may serve as models for nationwide implementation.

We observed a sharp increase in reported TB cases during 2004–2005. This may be attributed to the launch of the National Tuberculosis Information Management System (TBIMS) by the Chinese Center for Disease Control and Prevention (CDC) in 2005. This system enabled the online collection of information on all TB cases nationwide (20).

The TB mortality rate, although peaking in 2005, has been on the rise since 2015 (Figure 1B). In response, many relevant government departments in China conducted a joint investigation and released an action plan (2019–2022) (21), formulating comprehensive action goals and key prevention and control measures at the national level. However, predictive results indicate that China may still face challenges in achieving the World Health Organization’s 2030 tuberculosis control objectives. This disparity is due to two main factors: (1) poor control of drug-resistant TB in underdeveloped regions due to economic imbalances (22, 23), and (2) an uneven distribution of disease risk caused by differences in TB-related policies at the provincial level in China (24). In light of this situation, China needs to rely on the findings of cluster and predictive research, compare specific policies among provinces, and explore more effective and rational TB prevention strategies to achieve the goals set by the World Health Organization’s End TB Strategy.

Furthermore, we observed a significantly higher burden of PTB among older adult individuals (≥60 years old) in China compared to the younger population. Similar findings have been reported in several provincial and municipal areas across the country (25–28). This phenomenon can be attributed to two main factors. Firstly, policy measures play a crucial role. The Chinese government has led initiatives to implement health education programs on TB among children and adolescents in schools, maintain school environmental hygiene, and improve timely reporting mechanisms for TB cases (29). These measures effectively reduce the incidence of TB among the younger population. Secondly, the older adult population is more susceptible to TB due to their higher prevalence of underlying health conditions and weakened immune systems. This vulnerability increases their susceptibility to TB infection and raises the risk of reinfection (30–32). Current tuberculosis prevention and treatment policies should prioritize the older adult population by effectively raising TB awareness through educational outreach. Efforts should include BCG vaccine supplementation and the promotion of healthy lifestyles to boost immunity, ultimately helping to prevent TB transmission among older adults. Based on the results of cluster and time-series analyses, we observed regional characteristics in the spatiotemporal distribution of TB cases in China. Guizhou province, situated in mountainous areas, significantly lags behind Shanghai in terms of economic development. However, both Guizhou and Shanghai fall under Cluster (M) 5 regarding the trend of PTB mortality rates, showing a fluctuating downward trend within the range of 0.2/100,000 to 0.8/100,000. This fluctuating downward trend aligns with the global, European Region, and African Region trends reported in the Global Tuberculosis Report 2023 (1). Current research highlights that economic development levels and policy differences are crucial factors influencing the burden of PTB (33, 34). This suggests that the China Global Fund Tuberculosis Control Project, initiated and led by the Guizhou provincial government in 2003, played a pivotal role in controlling the spread of PTB within the province (35). The project proposes nine prevention and control measures to address the current situation and priorities of drug-resistant tuberculosis prevention and control in Guizhou Province. These measures encompass targeted strategies across the entire disease process, including screening, diagnosis, treatment, and prevention of drug-resistant TB. Additionally, practical preventive actions are recommended from the perspectives of patients, healthcare workers, and research experts. All of these efforts contribute to a more targeted approach to preventing and controlling the transmission of drug-resistant TB. Additionally, the time-series clustering of incidence rates in Tibet and Qinghai Province placed them in the same Cluster (I)3, indicating a continuous upward trend. This may be due to the fact that the residents in these regions are primarily herders with relatively low literacy levels and limited economic means, preventing them from accessing local healthcare services. This situation leads to the continued spread of PTB, impacting its prevention and control efforts (34). Tibet and Qinghai can draw on Guizhou Province’s TB prevention and control policies and update existing TB prevention and control measures by taking into account the specific characteristics of their populations. Local health departments should focus on the older adult population. Overall, the level of economic development significantly influences the clustering of TB morbidity and mortality. This influence stems from the direct impact of regional economic conditions on local income levels, healthcare services, and living conditions, which in turn causes differences in the number of TB diagnoses and cures. Particularly in economically underdeveloped regions, the prevalence of TB is exacerbated by a higher concentration of individuals with limited education. These individuals often lack awareness about tuberculosis and are less likely to seek treatment in more developed areas. Additionally, the local medical infrastructure in these regions is often inadequate, failing to effectively control the spread of the disease, thereby increasing TB incidence. Furthermore, people in economically disadvantaged areas are more likely to be in close contact with live animals and livestock, a known risk factor for TB transmission. This factor further contributes to the higher concentration of TB cases in these economically less developed regions to some extent.

From 2004 to 2018, the number of PTB cases in Xinjiang showed a gradual increase and was significantly higher than the national average. One possible reason for this trend is that Xinjiang has implemented a series of measures to screen for cases and control PTB outbreaks. By the end of 2010, Xinjiang’s DOTS coverage had reached 100%, and in 2013 it was the first province in the country to implement a new “trinity” model of TB control services. This model includes policies such as “centralized isolation treatment for infected patients and home treatment for non-infected patients, such as centralized medication + nutritious breakfast” (24). Although the number of TB cases appears to be increasing, this is actually a reflection of the continuous improvement in the TB reporting system, which should be maintained. Lessons can be drawn from Guizhou Province, which not only implements effective health policies within the province for TB prevention and control but also actively learns from more developed regions. For instance, Guizhou has invited experts from cities like Shanghai to provide scientific lectures and has adopted successful prevention and control strategies from Shanghai, such as enhancing the TB reporting system and ensuring timely follow-up for individuals infected with tuberculosis bacilli. National epidemiological surveys on tuberculosis should be conducted regularly to identify provinces with notable success in TB prevention and control. Promoting these best practices across provinces, increasing opportunities for interprovincial learning and exchange, and improving collaboration will ultimately enhance the overall efficiency of TB prevention and control efforts.

Using ARIMA model projections, we found an upward trend in TB mortality in mainland China since 2015. This trend is influenced by several factors. Firstly, there has been an increase in the burden of drug-resistant TB, with approximately 450,000 new cases of rifampicin-resistant TB reported in 2021 (36). Secondly, the impact of the COVID-19 pandemic led to the suspension of TB case reporting in 2021, potentially setting back TB control efforts by 8 years in achieving the goal of ending TB by 2030 (37). Pandemics have been shown to impact TB case reporting and treatment outcomes. Both TB and COVID-19 tests are more likely to yield false-negative results in a TB pandemic setting (38). Both diseases require better samples with independence to improve diagnostic precision and accuracy. Airborne and aerosol-based transmission are the primary modes of spread for both SARS-CoV-2 and *Mycobacterium tuberculosis*. Additionally, COVID-19 worsen the TB disease burden by increasing pulmonary disability and lung damage (39). To enhance positive case identification, numerous studies have conducted geospatial analyses to delineate the distribution of TB cases (40, 41). While TB is frequently prevalent in low-income areas and regions with inadequate sanitation, higher disease prevalence is often observed among individuals without stable residency status, including refugees, asylum seekers, and ordinary migrants (42). Infectious disease surveillance among incoming mobile populations should be enhanced, with consistent registration, screening, and testing for tuberculosis infection. The implementation of closure control policies due to the pandemic may have added complexity to the actual TB disease burden in mainland China, necessitating further investigation.

This research is subject to several potential limitations related to the dataset and clustering algorithms. Firstly, while the National Population and Health Scientific Data Sharing Platform provides high-quality data, it lacks comprehensive personal information about patients. This limitation prevents the analysis of certain demographic characteristics, such as gender and occupation, which are significant factors influencing TB distribution. Additionally, because the platform’s data is not updated to the most recent year, only retrospective studies can be conducted, introducing potential selection bias. Limitations in the inclusion criteria for new cases further constrain the study. On the algorithmic side, the DTW algorithm also presents limitations. It requires substantial computational resources, particularly when handling large datasets, and it focuses solely on the similarity between two sequences, without incorporating actual geographic information (16). These factors contribute to the study’s limitations. Firstly, PTB cases reported through the direct network reporting of infectious diseases may be subject to data bias due to variations in TB diagnostic capacity and healthcare provider practices. Secondly, our analysis was conducted at the provincial level, which may result in some level of data aggregation or disaggregation due to geographical scale effects. Thirdly, due to constraints in data collection, we were unable to explore in depth the etiological relationship between TB and risk confounding variables such as socioeconomic and environmental factors through multivariate analyses. However, despite these limitations, this study underscores the critical importance of PTB regulation. In the future, the prevention and treatment of tuberculosis will usher in a new phase of challenges in the post-epidemic era, necessitating both traditional, proven methods such as vaccination and the timely adaptation of prevention and treatment policies to more effectively achieve the World Health Organization’s (WHO) goal of eliminating tuberculosis. To support these objectives, research should prioritize two main areas: first, evaluating intervention effectiveness through health economics to identify the most impactful strategies, and second, leveraging advancements in artificial intelligence to develop efficient surveillance tools that can pinpoint high-risk areas and populations. This approach will enable a more responsive infectious disease system at all levels, facilitating the rapid detection of potential infection sources and the swift implementation of preventive and control measures to reduce transmission risks.

## 5 Conclusion

While China’s TB incidence continues to decline steadily, the burden remains significant, particularly due to regional inequalities and a concentration of cases among the older adult. These findings underscore the urgent need to enhance protective and preventive measures, along with intensified TB-related awareness campaigns and educational initiatives, to align with the World Health Organization’s milestones for TB control in China.

## Data Availability

The datasets presented in this study can be found in online repositories. The names of the repository/repositories and accession number(s) can be found in the article/Supplementary material.

## Footnotes

^1^ https://www.phsciencedata.cn/Share/index.jsp
