# Supplementary material for: Tuberculosis disease burden in China: a spatio-temporal clustering and prediction study
Source: Front Public Health. 2025 Jan 7;12:1436515. doi: 10.3389/fpubh.2024.1436515 (PMC11747482; doi:10.3389/fpubh.2024.1436515)
Supplement: Supplementary file 1 [file Data_Sheet_1.docx]

**Table S1** PTB incidence and mortality data and percentage change in mainland China and 31 provinces and municipalities, 2004-2018.

| Region | Incidence rate per 100,000（2004） | Incidence rate per 100,000（2018） | Percentage change for incidence rate | Mortality rate per 100,000（2004） | Mortality rate per 100,000（2018） | Percentage change for mortality rate |
| --- | --- | --- | --- | --- | --- | --- |
| Total | 74.64 | 59.27 | -20.59 | 0.11 | 0.23 | 105.35 |
| Beijing City | 48.36 | 30.43 | -37.07 | 0.12 | 0.09 | -27.96 |
| Tianjin City | 31.27 | 21.39 | -31.6 | 0.16 | 0.08 | -52.25 |
| Hebei Province | 55.78 | 39.31 | -29.53 | 0.04 | 0.10 | 171.26 |
| Shanxi Province | 55.13 | 35.03 | -36.47 | 0.04 | 0.08 | 92.81 |
| Inner mongolia Autonomous Region | 77.68 | 54.09 | -30.36 | 0.10 | 0.22 | 120.01 |
| Liaoning Province | 55.16 | 55.81 | 1.18 | 0.12 | 0.49 | 320.85 |
| Jilin Province | 85.62 | 47.43 | -44.6 | 0.10 | 0.14 | 34.41 |
| Heilongjiang Province | 102.93 | 65.23 | -36.63 | 0.51 | 0.59 | 16.63 |
| Shanghai City | 50.70 | 26.14 | -48.45 | 0.72 | 0.23 | -68.3 |
| Jiangsu Province | 63.73 | 31.02 | -51.33 | 0.10 | 0.10 | -4.51 |
| Zhejiang Province | 91.34 | 45.26 | -50.45 | 0.19 | 0.21 | 10.47 |
| Anhui Province | 53.41 | 51.08 | -4.37 | 0.04 | 0.17 | 379.22 |
| Fujian Province | 80.89 | 45.24 | -44.08 | 0.06 | 0.08 | 44.47 |
| Jiangxi Province | 87.58 | 70.83 | -19.12 | 0.07 | 0.15 | 113.15 |
| Shandong Province | 33.42 | 28.12 | -15.87 | 0.02 | 0.10 | 349.44 |
| Henan Province | 69.01 | 55.16 | -20.07 | 0.04 | 0.10 | 124.51 |
| Hubei Province | 99.44 | 63.49 | -36.15 | 0.05 | 0.16 | 196.15 |
| Hunan Province | 64.20 | 78.75 | 22.66 | 0.05 | 0.19 | 272.28 |
| Guangdong Province | 75.77 | 64.81 | -14.46 | 0.13 | 0.12 | -8.63 |
| Guangxi Zhuang Autonomous Region | 119.62 | 82.82 | -30.77 | 0.22 | 0.45 | 110.17 |
| Hainan Province | 123.21 | 85.05 | -30.97 | 0.07 | 0.19 | 157.27 |
| Chongqing City | 127.49 | 73.37 | -42.45 | 0.11 | 0.41 | 280.2 |
| Sichuan Province | 85.04 | 57.34 | -32.58 | 0.10 | 0.19 | 81.09 |
| Guizhou Province | 122.23 | 114.06 | -6.69 | 0.17 | 0.30 | 74.14 |
| Yunnan Province | 53.67 | 59.61 | 11.08 | 0.17 | 0.21 | 19.73 |
| Tibet Autonomous Region | 74.32 | 166.66 | 124.24 | 0.11 | 0.53 | 364.77 |
| Shannxi Province | 91.47 | 55.90 | -38.88 | 0.17 | 0.18 | 9.45 |
| Gansu Province | 76.26 | 38.71 | -49.24 | 0.09 | 0.05 | -41.93 |
| Qinghai Province | 71.78 | 140.33 | 95.5 | 0.07 | 0.32 | 328.31 |
| Ningxia Hui Autonomous Region | 66.08 | 36.73 | -44.42 | 0.00 | 0.13 | NA |
| Xinjiang Uygur Autonomous Region | 142.02 | 304.94 | 114.72 | 0.17 | 2.47 | 1336.4 |

**Table S2** PTB incidence and mortality annual percentage changes (APCs) in mainland China and 31 provinces and municipalities, 2004-2018.

| Region | Incidence rate | | | Mortality rate | | |
| --- | --- | --- | --- | --- | --- | --- |
|  | APC_1_ | APC_2_ | APC_3_ | APC_1_ | APC_2_ | APC_3_ |
| Total | 9.0 (1.5, 17.0) | -5.3 (-7.4, -3.1) | -2.9 (-3.8, -1.9) | 50.7 (8.2, 109.8) | -6.2 (-9.5, -2.8) | 11.5 (-5.5, 31.6) |
| Beijing City | 28.3 (11.4,47.4) | -7.4 (-10.2, -4.6) | -0.3 (-8.7, 8.9) | 4.9 (-6.8, 18.0) | -16.9 (-65.6, 101.1) | 4.1 (-21.3, 37.7) |
| Tianjin City | 6.6 (-12.5, 29.8) | -6.9 (-8.9, -4.9) | 3.6 (-6.1, 14.3) | -20.6 (-47.7, 20.7) | 38.3 (-63.2,419.3) | -19.4 (-32.5, -3.8) |
| Hebei Province | 3.9 (1.6, 6.3) | -6.3 (-12.8, 0.7) | -4.3 (-5.3, -3.4) | 53.6 (-26.7, 222.0) | -11.2 (-18.1, -3.7) | 41.5 (-2.3, 104.8) |
| Shanxi Province | 11.7 (-1.2, 26.4) | -2.9 (-8.7, 3.3) | -8.7 (-9.9, -7.4) | 66.0 (-22.2, 254.6) | -12.7 (-23.2, -0.8) | 14.7 (-3.2, 35.9) |
| Inner mongolia Autonomous Region | 10.0 (-5.8, 28.6) | -8.0 (-9.5, -6.4) | 2.9 (-4.8, 11.3) | 76.2 (-21.5, 295.4) | -15.5 (-21.5, -9.1) | 55.2 (-30.8, 248.4) |
| Liaoning Province | 2.8 (1.4, 4.1) | -6.7 (-12.2, -0.9) | 5.5 (-6.5, 19.0) | 35.7 (-14.8, 116.2) | -5.4 (-14.5,4.8) | 51.5 (-40.3, 284.6) |
| Jilin Province | -5.7 (-15.2,4.8) | 2.0 (-17.4, 25.9) | -7.3(-9.4, -5.2) | 8.0 (-4.0, 21.4) | -31.0 (-71.3, 65.8) | 12.0(-15.2,47.8) |
| Heilongjiang Province | -3.2 (-5.5, -0.8) | -0.6 (-20.3, 23.9) | -9.2 (-18.7, 1.3) | -1.6 (-6.1,3.0) | -5.0 (-42.6, 57.2) | 21.5 (-26.6, 101.1) |
| Shanghai City | -15.4 (-23.5, -6.5) | 1.7 (-8.0, 12.4) | -3.8 (-4.7, -2.9) | -18.7 (-42.8, 15.5) | 9.1 (-23.2, 55.0) | -11 (-13.8, -8.1) |
| Jiangsu Province | 0.9 (-4.8, 6.9) | -6.1 (-6.7, -5.5) | -7.5 (-10.1, -4.7) | 56.8 (-10.9, 176.1) | -14.0(-28.1,2.8) | -4.5 (-11.5, 3.0) |
| Zhejiang Province | -2.1 (-5.6, 1.6) | -9.2 (-12.4, -5.8) | -3.7 (-4.7, -2.8) | 2.5 (-11.4, 18.6) | -11.0 (-62.5, 110.7) | 5.7 (-12.8, 28.2) |
| Anhui Province | 28.3 (11.4,47.7) | -9.3 (-15.5, -2.7) | -2.8 (-4.3, -1.3) | 87.2 (-6.9, 276.7) | 0.0 (-8.9, 9.8) | 2.5 (-17.8, 27.8) |
| Fujian Province | -0.9 (-7.4, 6.1) | -15.9 (-32.1,4.3) | -2.4 (-5.2, 0.4) | 97.4 (2.9, 278.7) | -9.8 (-22.0, 4.4) | -5.2 (-15.1,5.8) |
| Jiangxi Province | 3.0 (-7.6, 14.8) | -5.4 (-7.1, -3.6) | -0.2 (-2.6, 2.3) | 83.3 (15.5, 190.9) | -15.7 (-20.8, -10.4) | 19.1 (2.9, 37.9) |
| Shandong Province | 11.7 (3.0, 21.1) | -0.2 (-8.0, 8.2) | -4.9 (-5.6, -4.2) | 131.7 (63.4, 228.6) | -6.9 (-11.1, -2.4) | 4.4 (-6.5, 16.6) |
| Henan Province | 17.8 (7.8, 28.7) | -8.6 (-12.6, -4.5) | -3.6 (-4.5, -2.6) | 97.3 (41.3, 175.5) | -2.6 (-30.3, 35.9) | -9.0 (-11.7, -6.2) |
| Hubei Province | 2.4 (-6.1, 11.8) | -7.5 (-22.4, 10.2) | -3.5 (-5.3, -1.7) | 69.0 (-10.1,217.9) | -5.9 (-13.5, 2.4) | 7.6 (-11.9, 31.3) |
| Hunan Province | 21.5 (7.5, 37.4) | -3.3 (-9.0, 2.9) | -2.2 (-3.5, -0.9) | 134.7 (56.4, 252.3) | -9.5 (-13.4, -5.4) | 7.8 (-12.0, 32.1) |
| Guangdong Province | 6.3(1.7, 11.2) | -11.9 (-27.9, 7.5) | -1.7 (-5.0, 1.6) | 16.3 (-9.1,48.8) | -11.3 (-16.0, -6.4) | 22.7 (-25.0, 100.8) |
| Guangxi Zhuang Autonomous Region | 1.7 (-8.5, 13.0) | -8.9 (-26.3, 12.5) | -2.0 (-4.2, 0.3) | 75.3 (-22.9, 298.5) | -11.7 (-19.3, -3.5) | 36.3 (-9.6, 105.6) |
| Hainan Province | 5.0 (-2.0, 12.4) | -9.4 (-21.1,4.0) | -3.2 (-4.6, -1.7) | 96.0 (-8.8, 321.4) | -7.8 (-14.1, -1.2) | 4.0 (-51.6, 123.5) |
| Chongqing City | -1.0 (-11.3, 10.5) | -12.0 (-29.4, 9.6) | -2.7 (-5.0, -0.4) | 63.8 (-1.1, 171.4) | -4 (-14.2, 7.5) | 6.0 (-2.7, 15.4) |
| Sichuan Province | 8.9 (1.6, 16.7) | -6.2 (-9.4, -2.9) | -4.1 (-4.8, -3.4) | 60.4 (3.8, 148.0) | -6.3 (-10.6, -1.7) | 4.1 (-16.2, 29.5) |
| Guizhou Province | 8.2 (1.5, 15.3) | -9.6 (-26.1, 10.6) | -1.1 (-3.7, 1.6) | 43.0 (-26.5, 178.4) | 6.0 (-8.7, 23.0) | -12.2 (-21.5, -1.7) |
| Yunnan Province | 4.1 (-5.4, 14.6) | -1.8 (-2.8, -0.7) | 3.0 (-1.8, 8.1) | 34.6 (-25.1, 141.9) | -8.1 (-12.9, -3.1) | 14.1 (-36.5, 105.1) |
| Tibet Autonomous Region | -0.6 (-8.5, 8.0) | 15.7 (-11.0, 50.4) | 3.0 (-0.6, 6.7) | 27.5 (10.5, 47.2) | -40.6 (-84.0, 120.5) | 29.0 (-33.1, 148.7) |
| Shannxi Province | -2.9 (-15.1, 11.0) | -5.8 (-7.5, -4.1) | 0.1 (-4.0, 4.5) | 2.6 (-27.4,45.1) | -17.6 (-22.3, -12.7) | 28.2 (18.7, 38.6) |
| Gansu Province | 13.4 (3.3, 24.5) | -13.8 (-35.9, 15.9) | -8.6 (-12.1, -4.9) | 88.3 (-11.0,298.1) | -8.4 (-14.5, -1.9) | -38.7 (-71.0,29.7) |
| Qinghai Province | 4.3 (-11.8, 23.3) | 1.0 (-27.7,41.2) | 5.7 (1.9, 9.7) | 22.3 (-7.6, 61.9) | -27.1 (-79.2, 155.5) | 14.9 (-7.0, 42.0) |
| Ningxia Hui Autonomous Region | 7.1 (-15.8, 36.2) | -7.7 (-14.5, -0.4) | -3.4 (-6.5, -0.2) | -0.5 (-28.5, 38.5) | -10.5 (-68.5, 154.3) | 8.7 (-8.9, 29.7) |
| Xinjiang Uygur Autonomous Region | 12.9 (-13.5, 47.2) | -1.5 (-3.8, 1.0) | 31.1 (0.5, 71.1) | 130.8 (27.3, 318.5) | -7.8 (-19.3, 5.3) | 20.0 (8.5, 32.7) |

**Table S3** Accuracy test results of 5 prediction models (for incidence rate)

| Model | mae | mape | mase | smape | rmse | rsq |
| --- | --- | --- | --- | --- | --- | --- |
| ARIMA | 0.20 | 4.04 | 0.55 | 3.93 | 0.24 | 0.86 |
| PROPHET | 0.43 | 9.45 | 1.19 | 10.40 | 0.57 | 0.90 |
| GLMNET | 0.38 | 7.71 | 1.07 | 7.86 | 0.44 | 0.87 |
| RANDOMFOREST | 0.58 | 12.09 | 1.62 | 11.27 | 0.63 | 0.77 |
| PROPHET BOOST | 0.21 | 4.20 | 0.59 | 4.13 | 0.25 | 0.90 |

**Table S4** Accuracy test results of 5 prediction models (for mortality rate)

| Model | mae | mape | mase | smape | rmse | rsq |
| --- | --- | --- | --- | --- | --- | --- |
| ARIMA | 0 | 6.53 | 0.70 | 6.69 | 0 | 0.30 |
| PROPHET | 0 | 26.08 | 2.79 | 31.28 | 0 | 0.06 |
| GLMNET | 0 | 7.75 | 0.85 | 8.15 | 0 | NA |
| RANDOMFOREST | 0 | 15.83 | 1.71 | 17.40 | 0 | 0.08 |
| PROPHET BOOST | 0 | 18.46 | 1.95 | 16.46 | 0 | 0.13 |
