# Supplementary material for: Tuberculosis disease burden in China: a spatio-temporal clustering and prediction study
Source: Front Public Health. 2025 Jan 7;12:1436515. doi: 10.3389/fpubh.2024.1436515 (PMC11747482; doi:10.3389/fpubh.2024.1436515)
Supplement: Supplementary file 2 [file Data_Sheet_2.docx]

**Part 1: Fig. S1. & S2.** Two “forest maps” of tuberculosis disease burden in mainland China, 2004-2018.

**
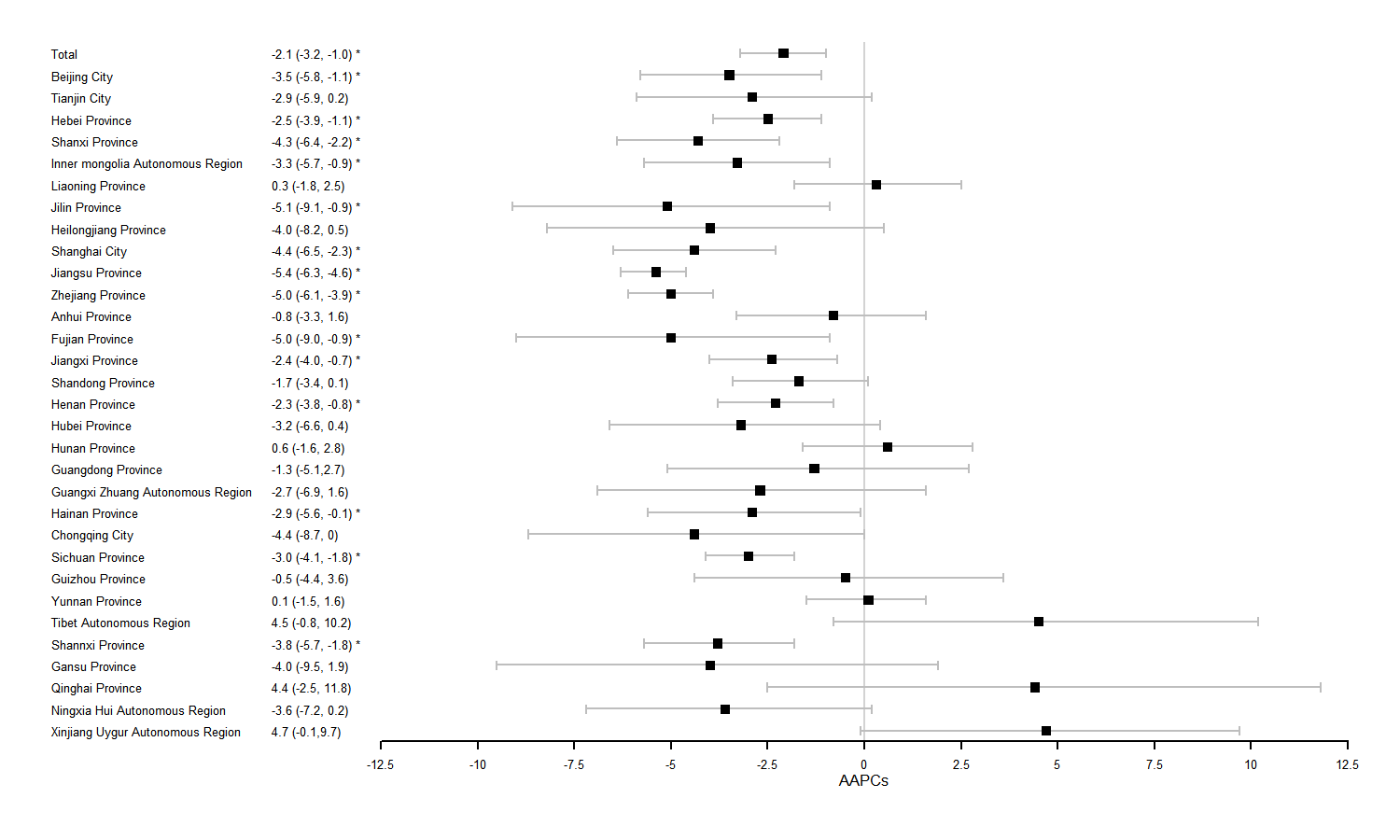
**

**Fig. S1.** A “forest map” of tuberculosis incidence rates in mainland China, 2004-2018.


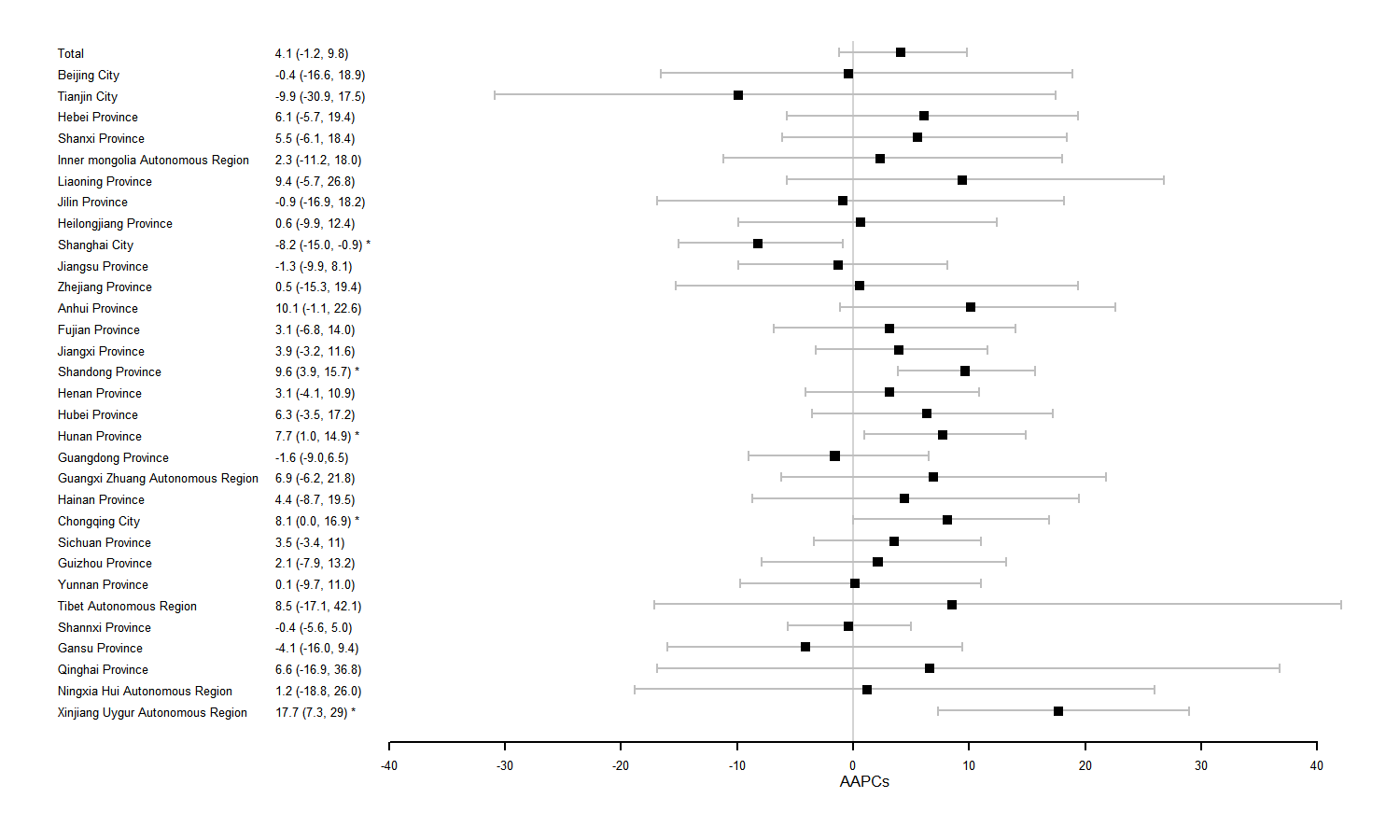
**Fig. S2.** A “forest map” of tuberculosis mortality rates in mainland China, 2004-2018.

**Part 2: Fig. S3-Fig. S9** Time-series plot of tuberculosis incidence rates in 31 provinces in mainland China (by clustering results).


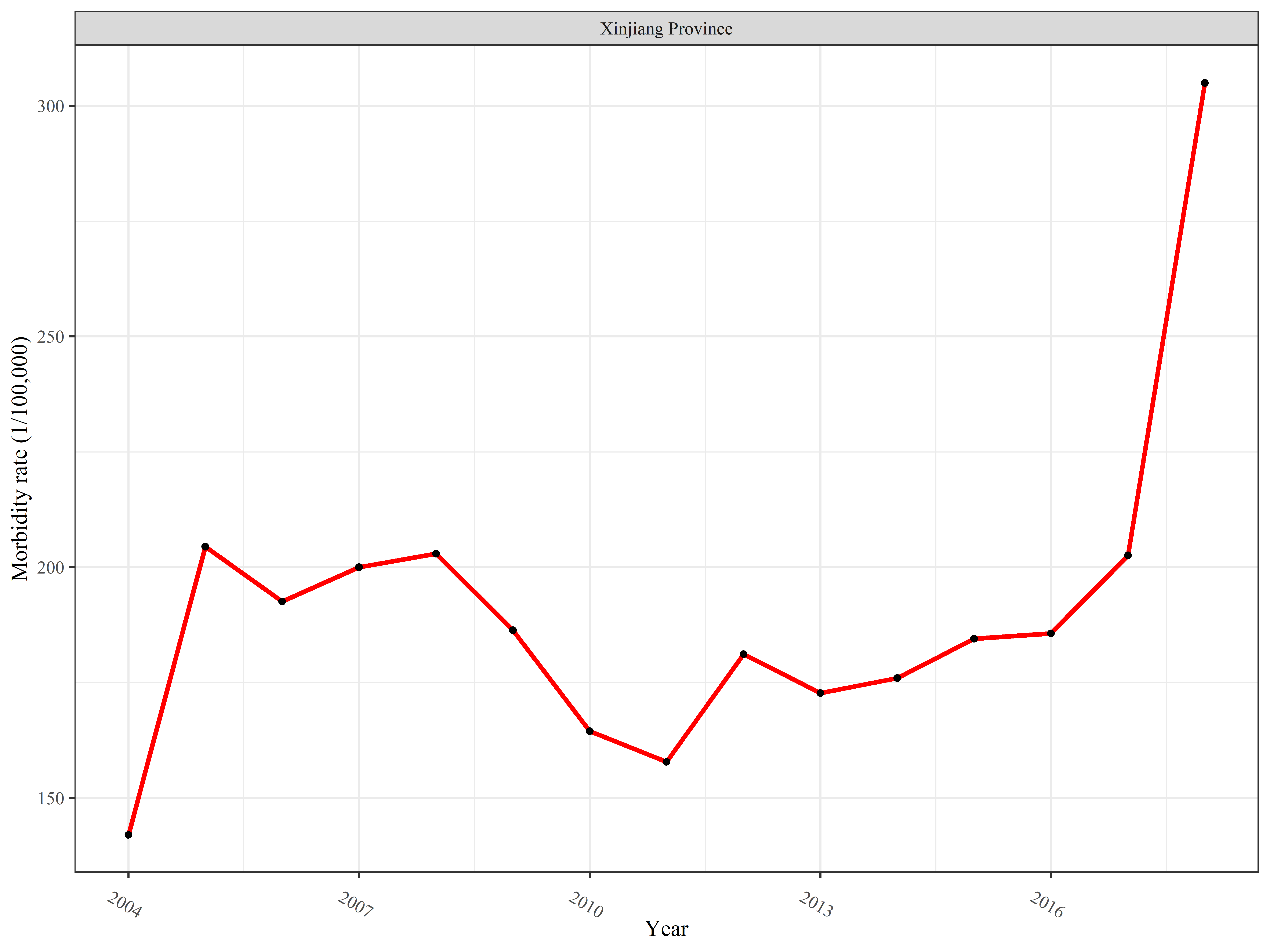


**Fig. S3.** Time-series plot of tuberculosis incidence (Category I).


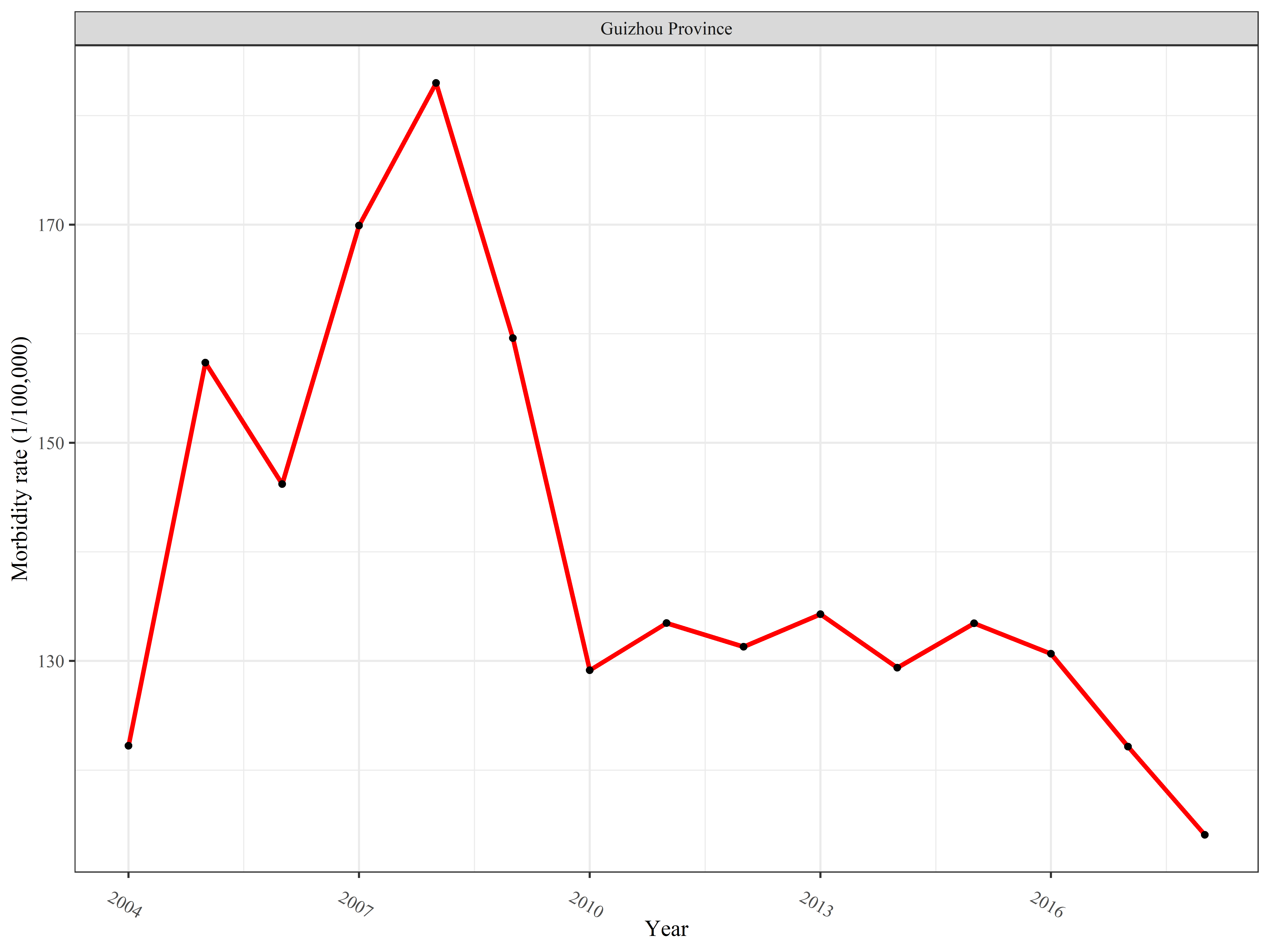


**Fig. S4.** Time-series plot of tuberculosis incidence (Category Ⅱ).

**
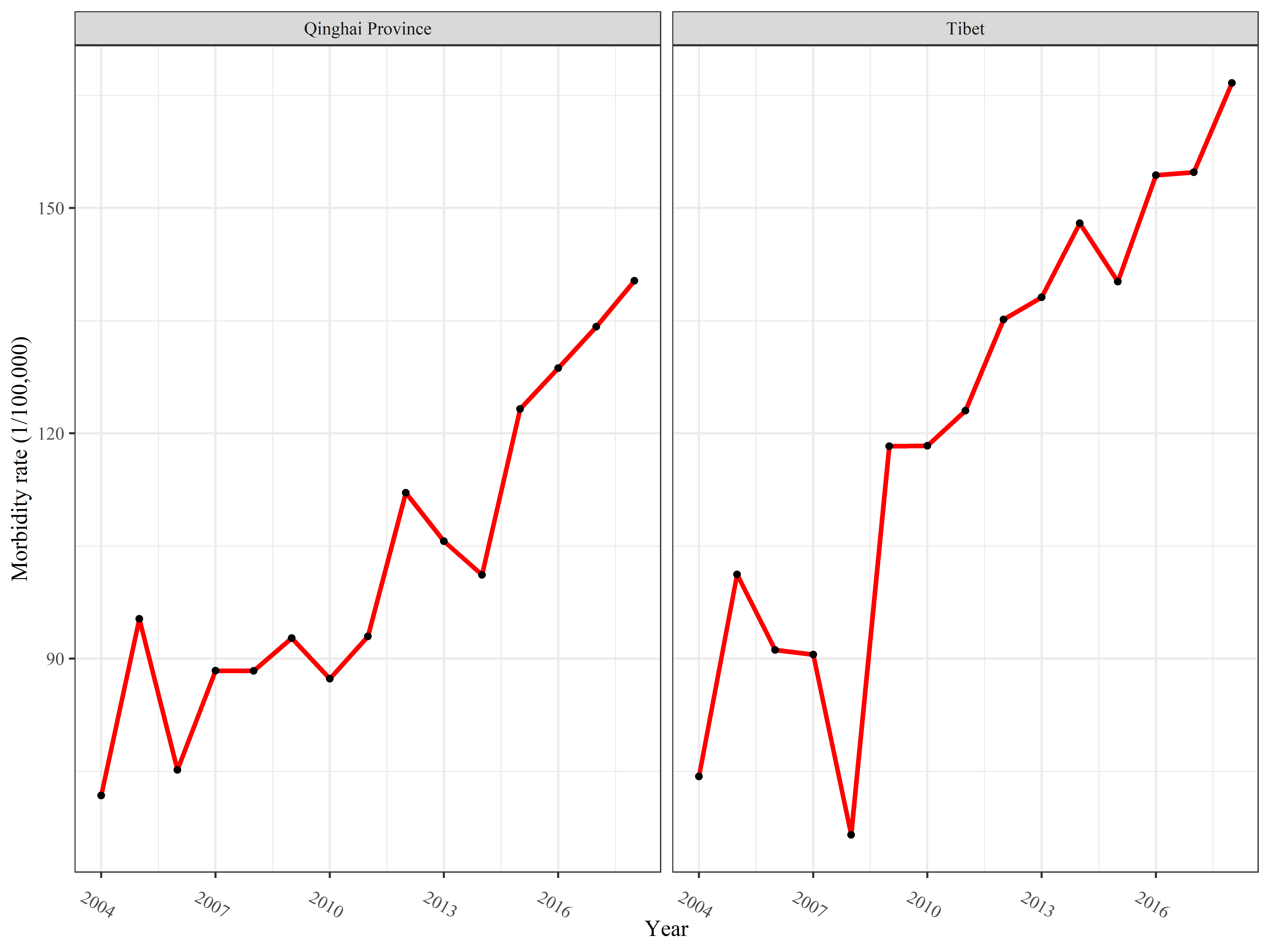
**

**Fig. S5.** Time-series plot of tuberculosis incidence (Category Ⅲ).


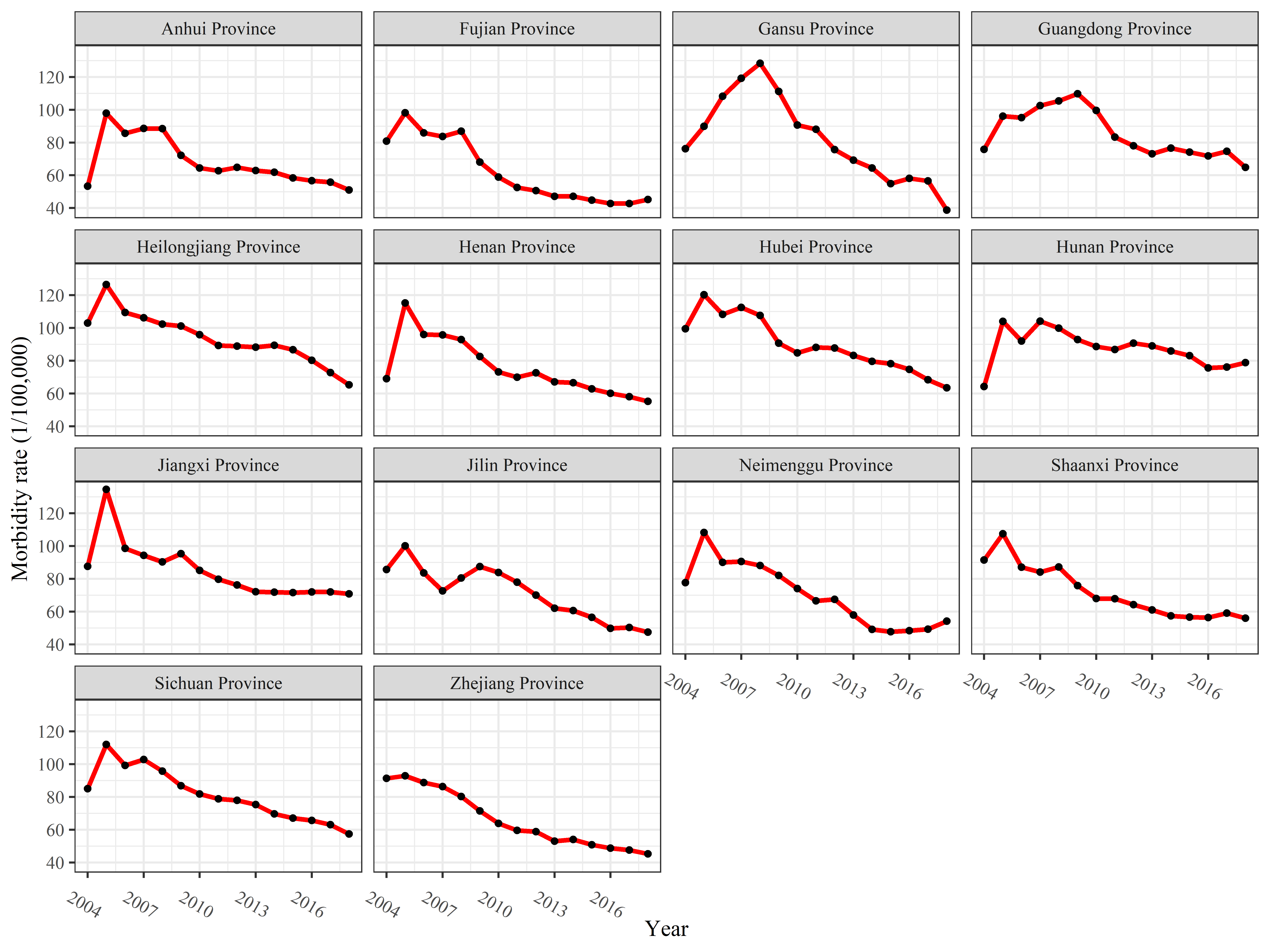


**Fig. S6.** Time-series plot of tuberculosis incidence (Category Ⅳ).

**
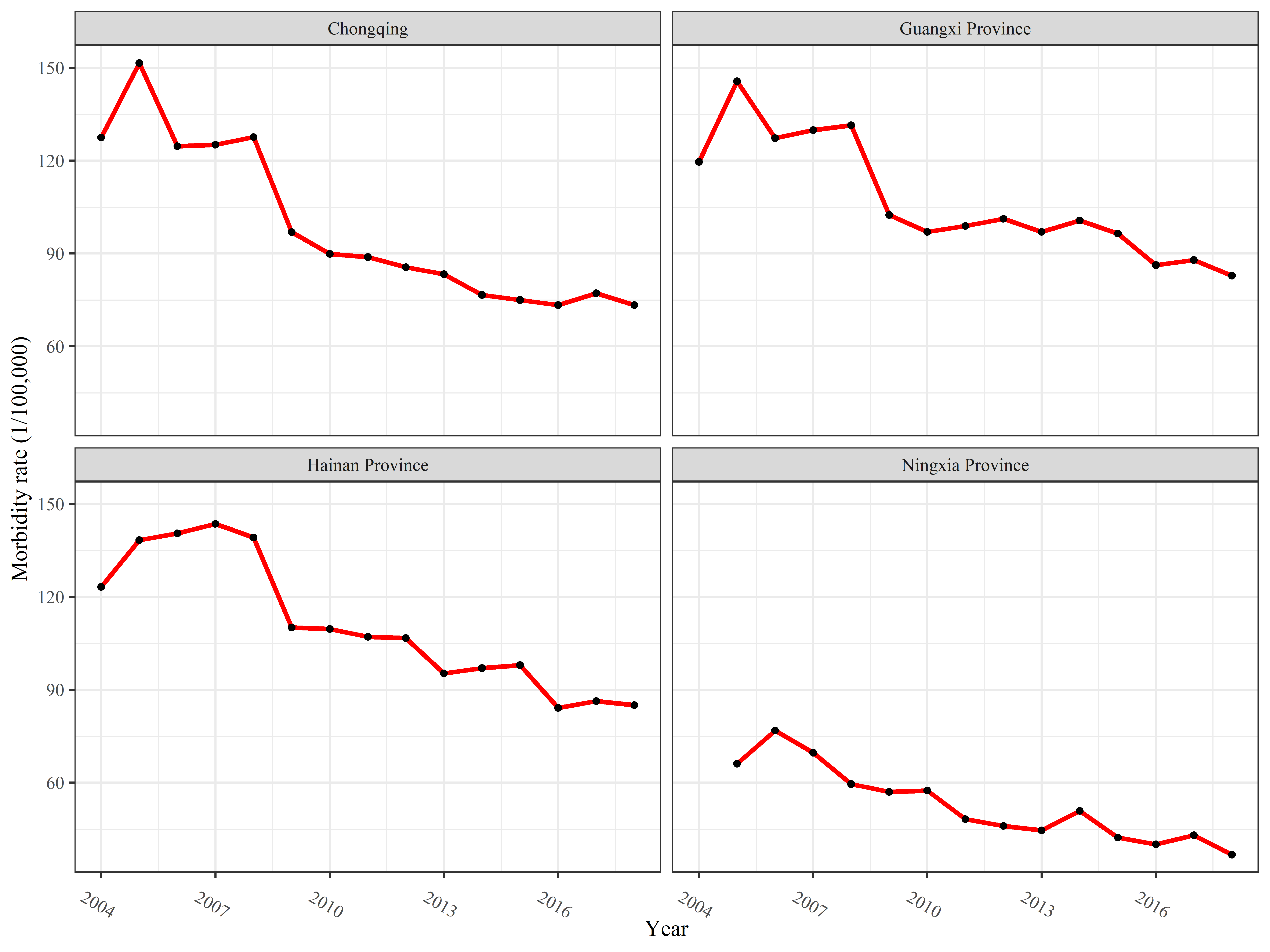
**

**Fig. S7.** Time-series plot of tuberculosis incidence (Category Ⅴ).

**
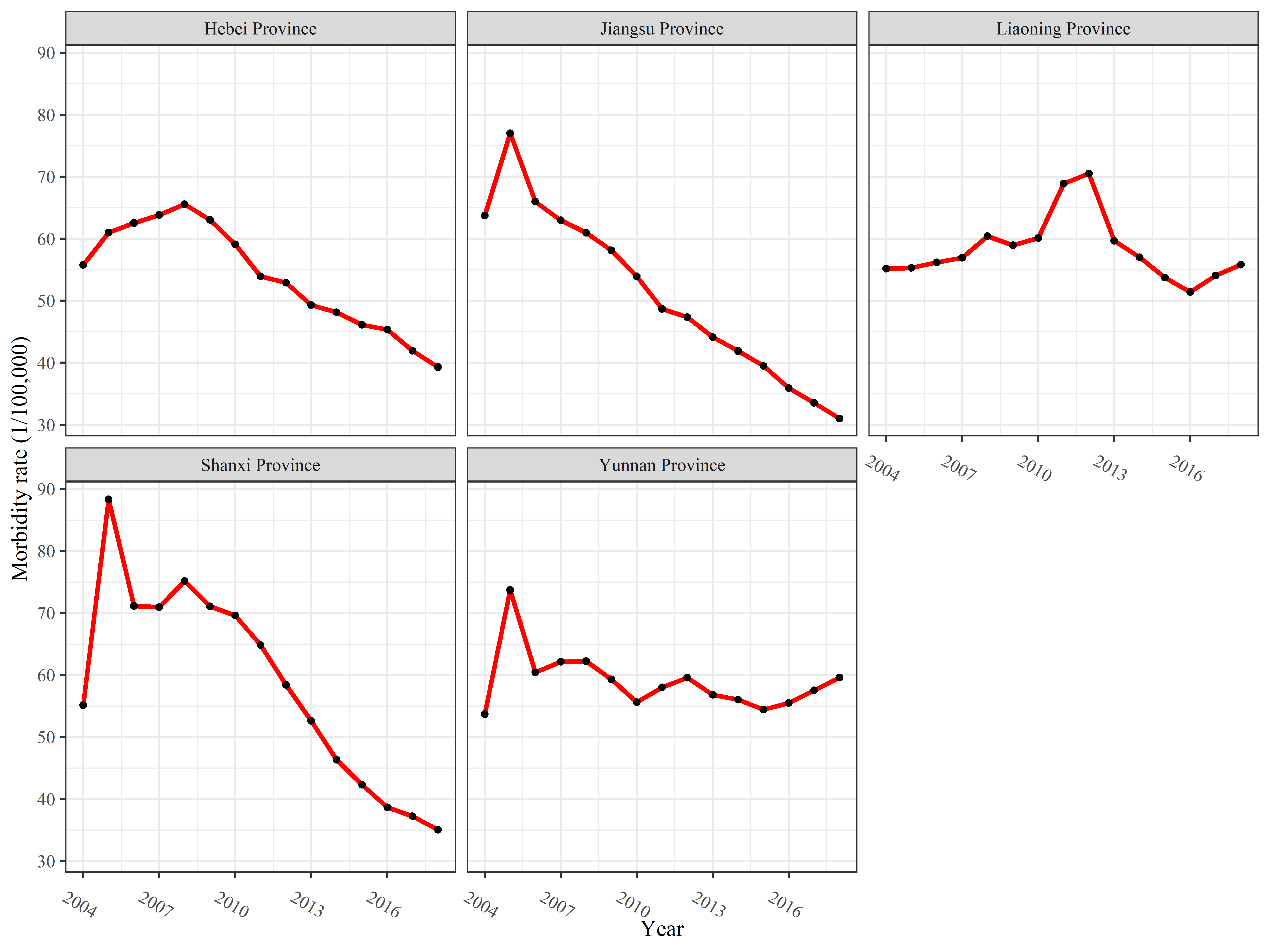
**

**Fig. S8.** Time-series plot of tuberculosis incidence (Category Ⅵ).

**
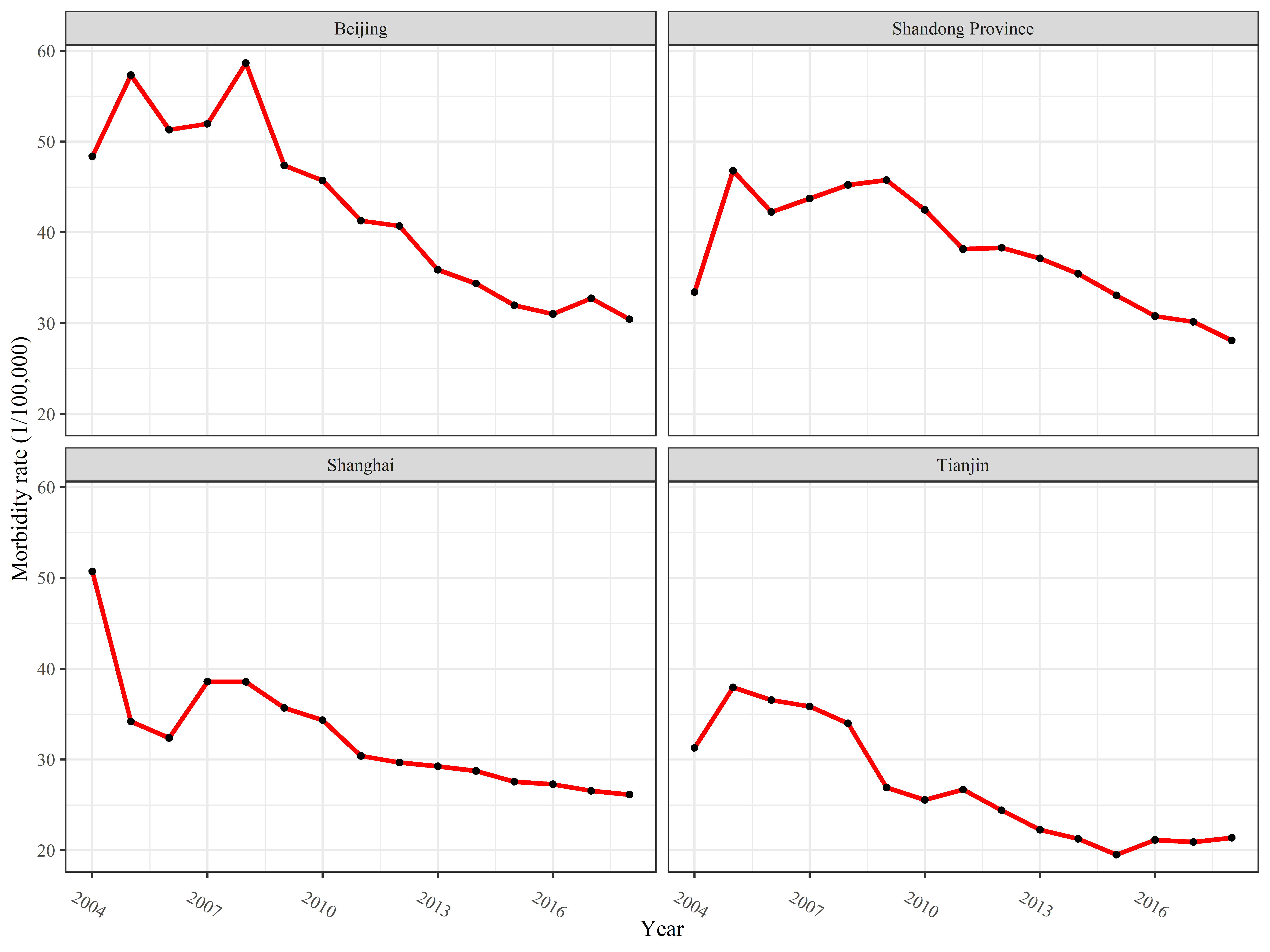
**

**Fig. S9.** Time-series plot of tuberculosis incidence (Category Ⅶ).

**Part 3: Fig. S10-Fig. S15** Time-series plot of tuberculosis mortality rates in 31 provinces in mainland China (by clustering results).


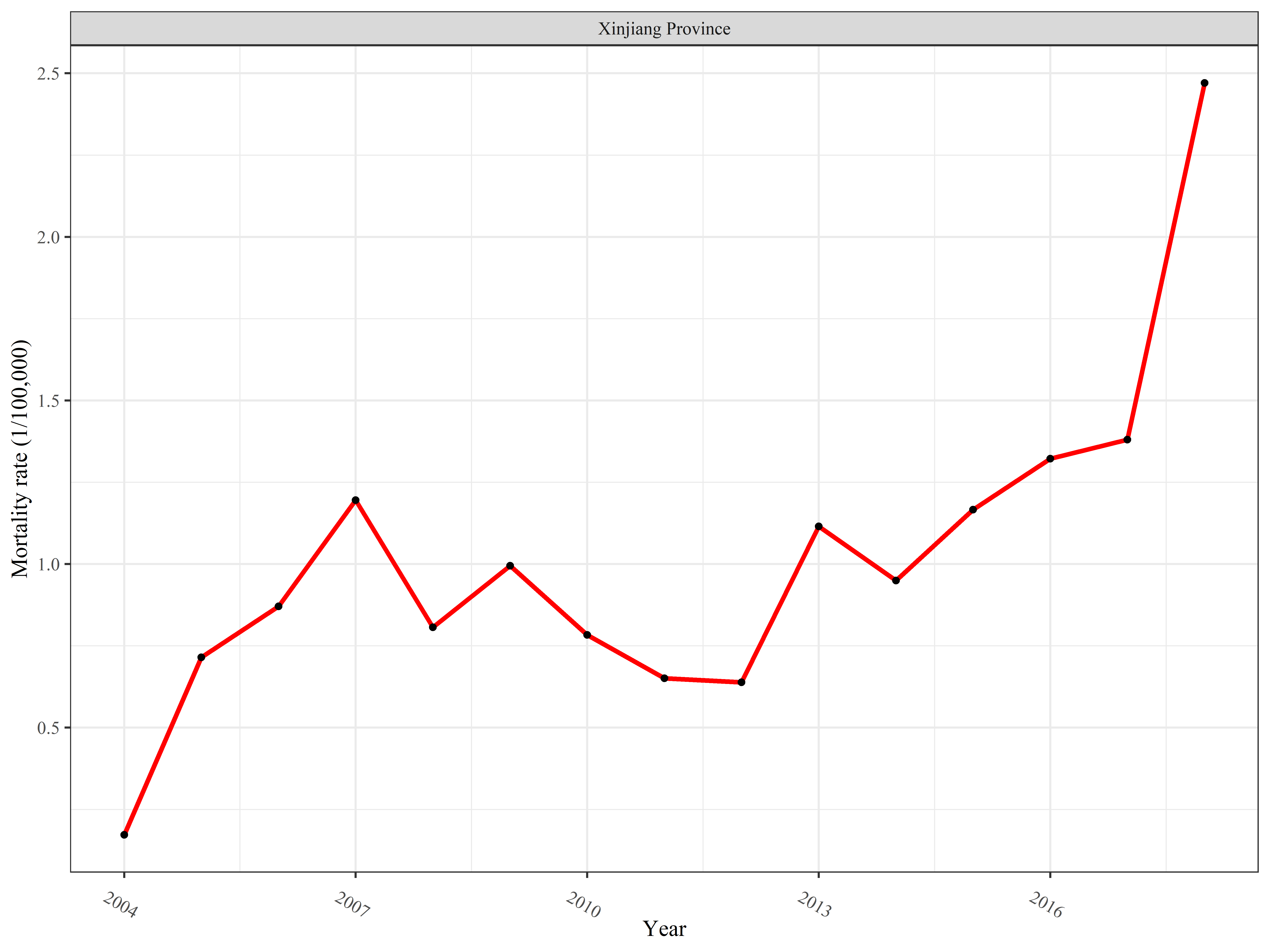


**Fig. S10.** Time-series plot of tuberculosis incidence (Category Ⅰ).

**
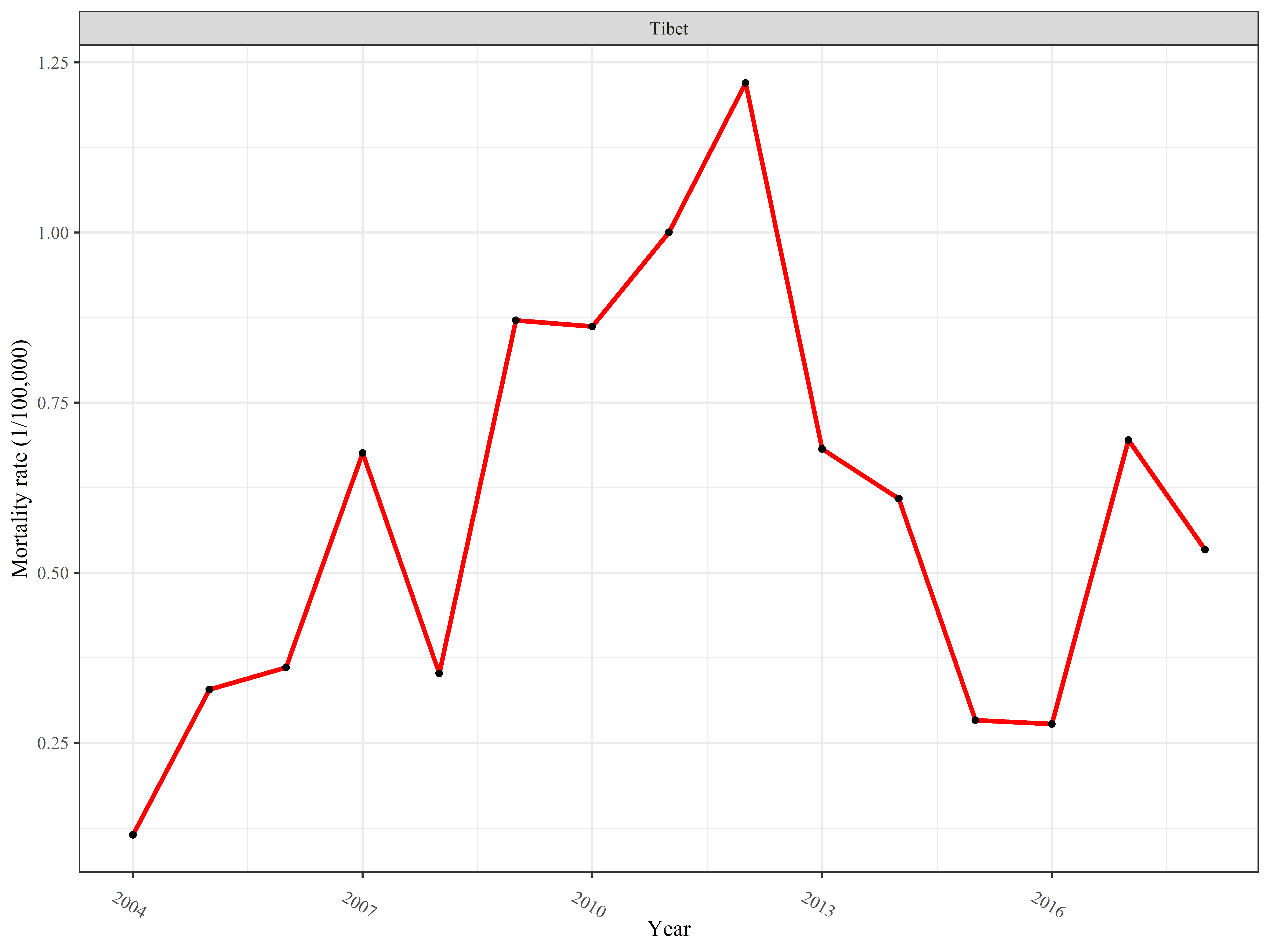
**

**Fig. S11.** Time-series plot of tuberculosis incidence (Category Ⅱ).

**
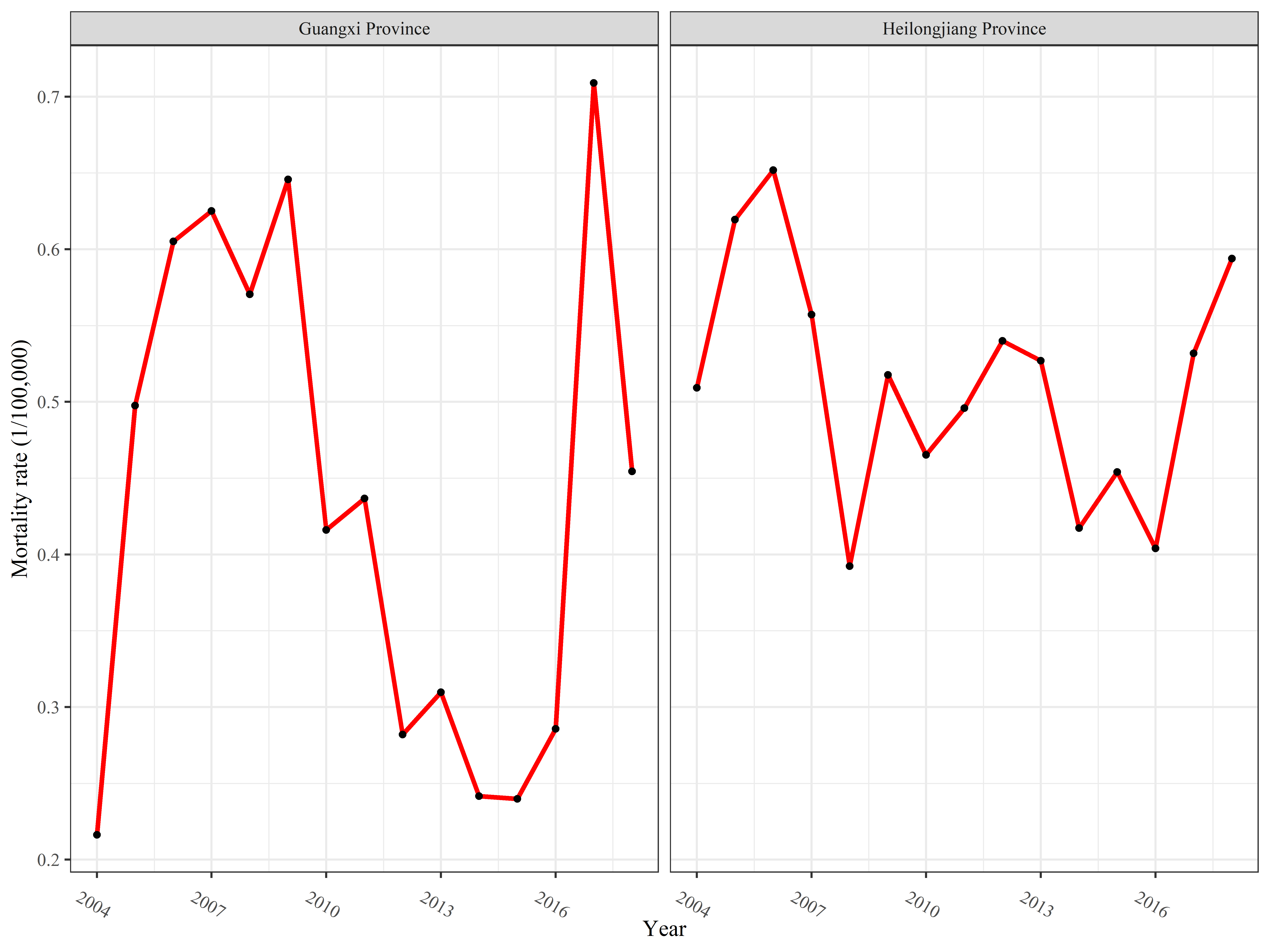
**

**Fig. S12.** Time-series plot of tuberculosis incidence (Category Ⅲ).

**
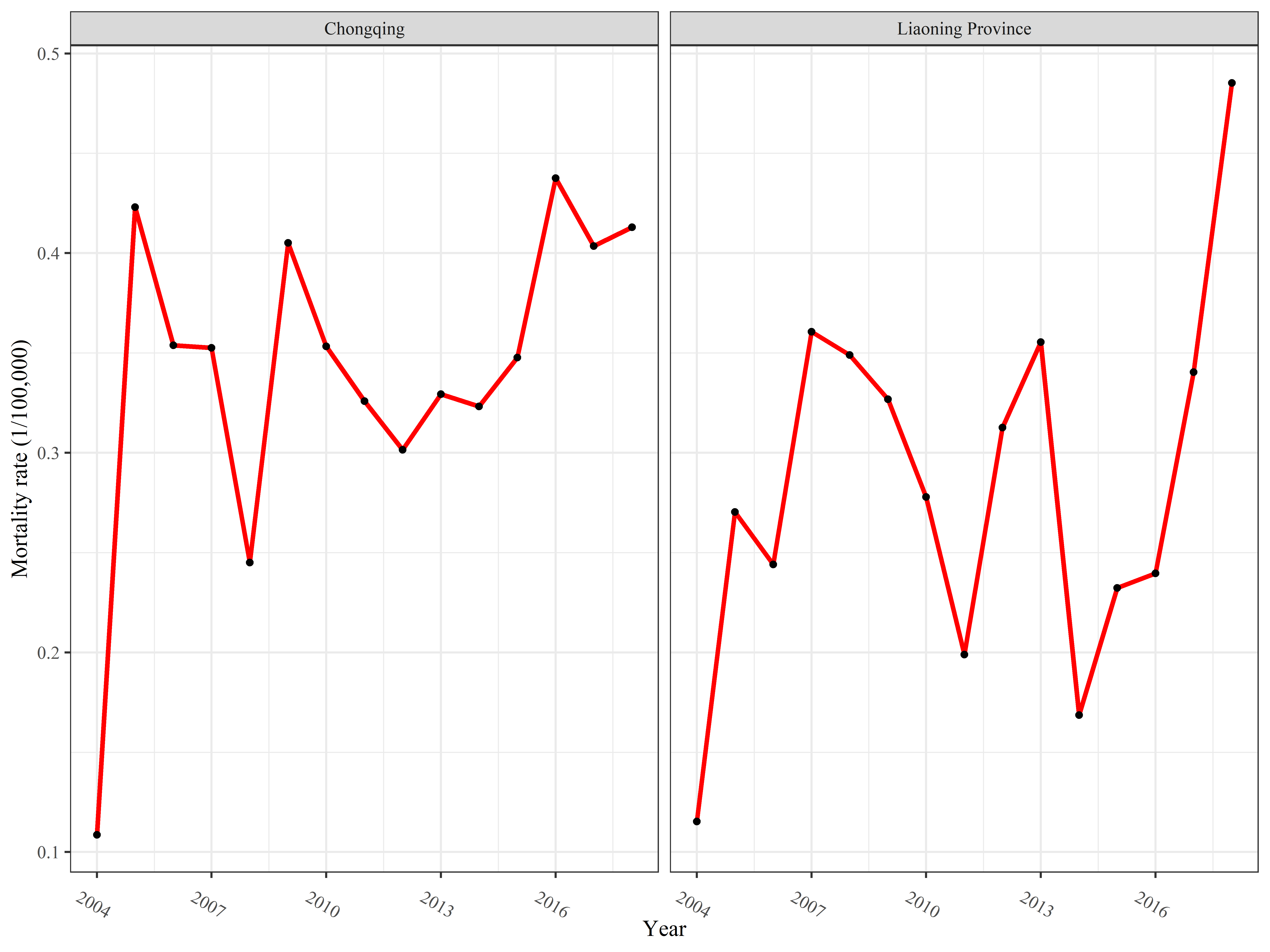
**

**Fig. S13.** Time-series plot of tuberculosis incidence (Category Ⅳ).

**
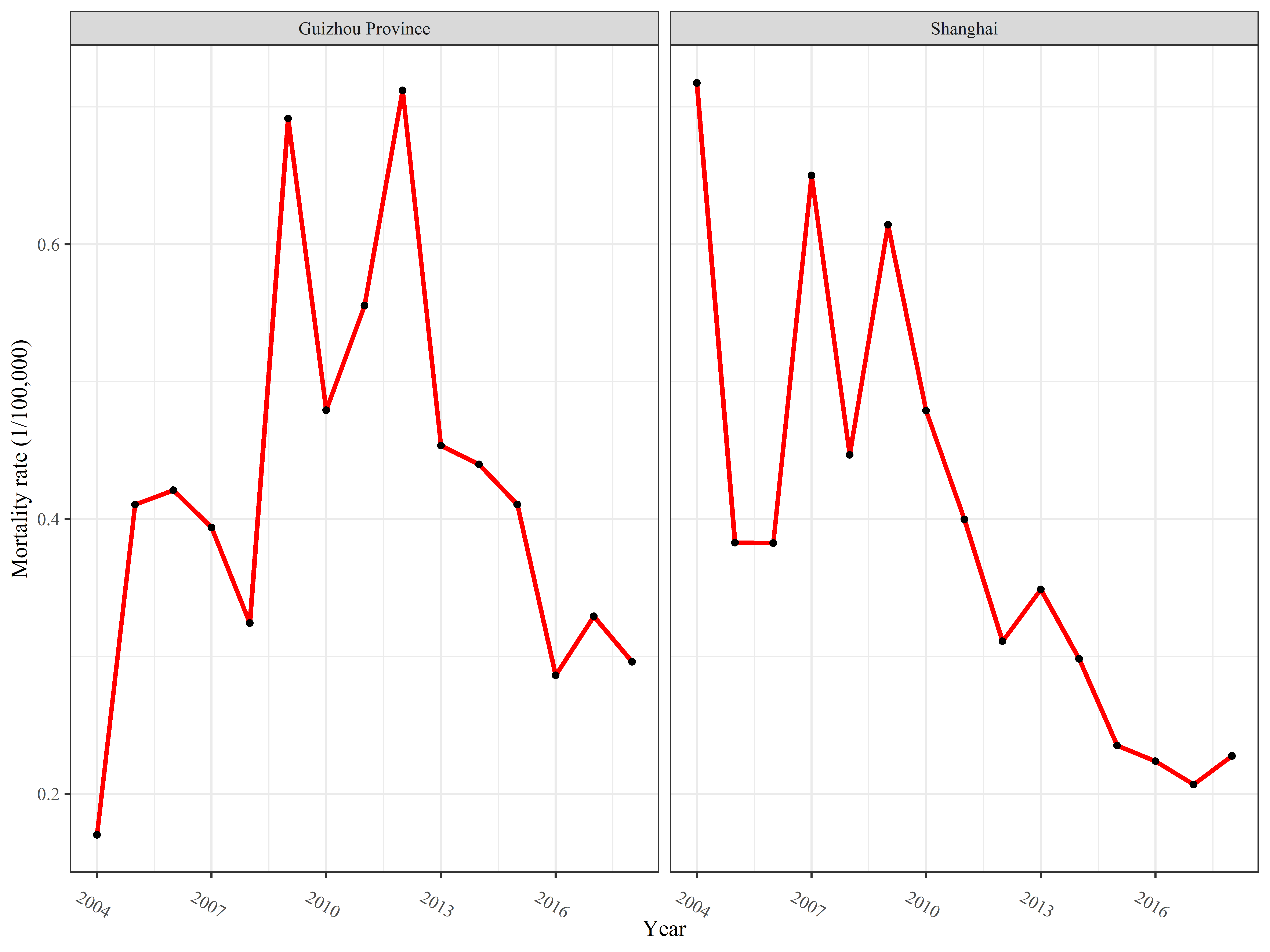
**

**Fig. S14.** Time-series plot of tuberculosis incidence (Category Ⅴ).

**
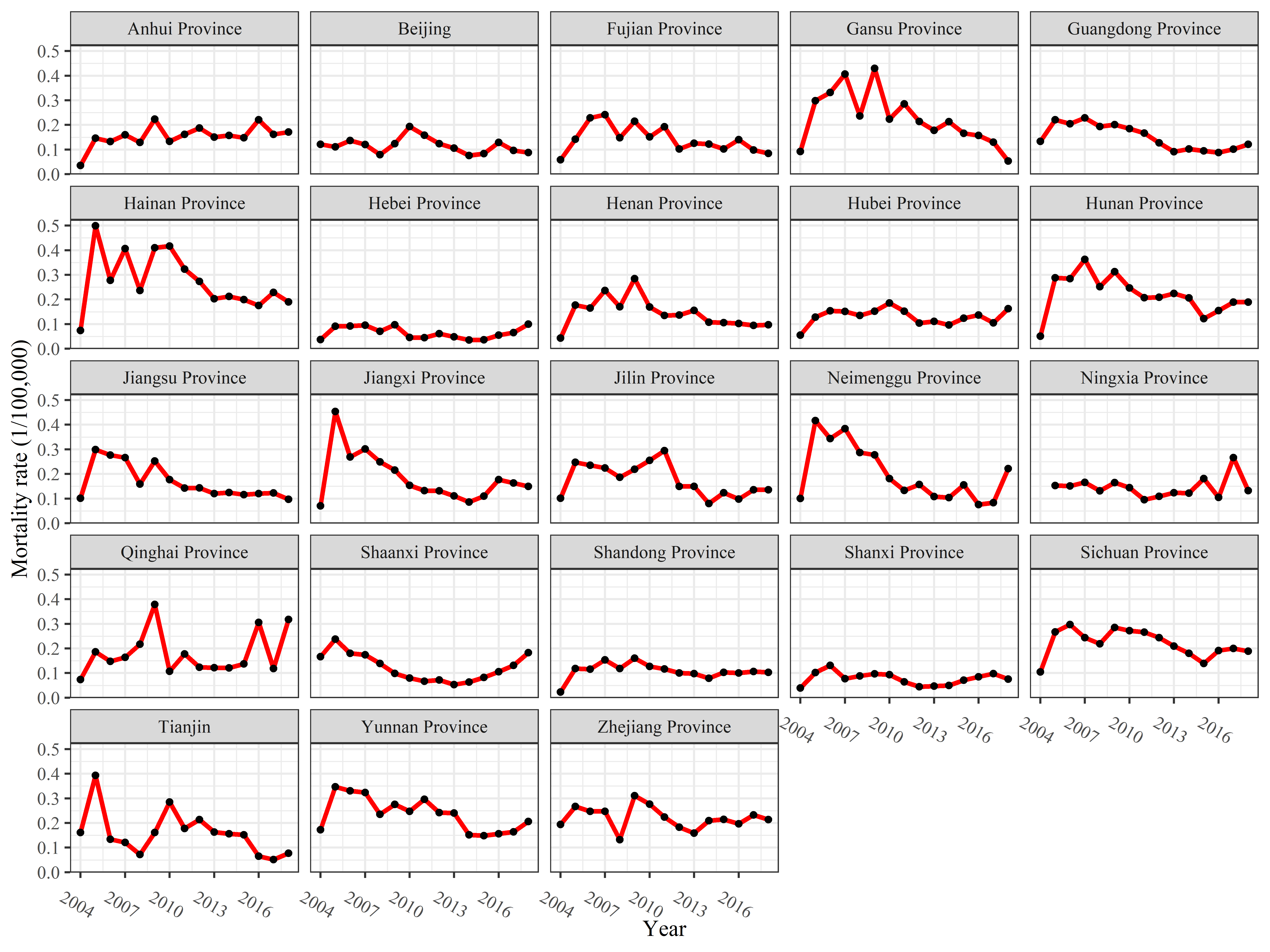
**

**Fig. S15.** Time-series plot of tuberculosis incidence (Category Ⅵ).

**Part 4: Fig. S16-Fig. S17** Tuberculosis burden by month, quarter, and year for 31 provinces in mainland China (by predictive study results).

**
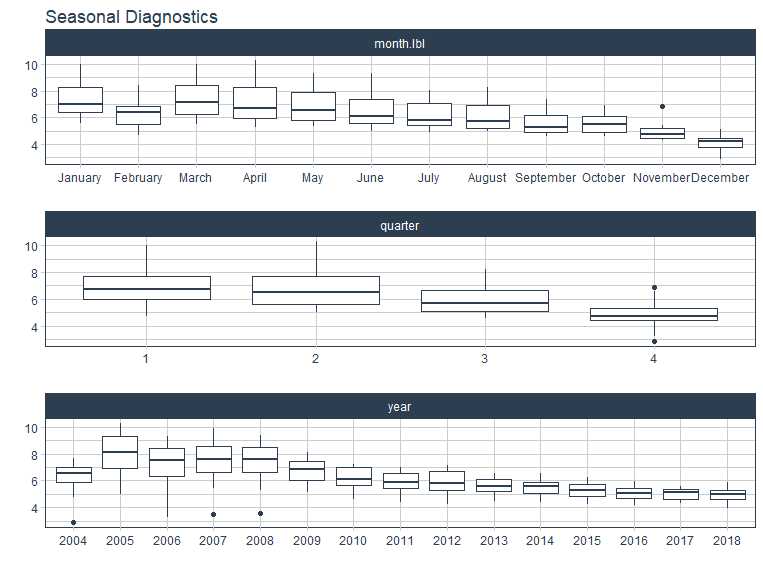
**

**Fig. S16.** Tuberculosis incidence rates by month, quarter, and year for 31 provinces in mainland China.


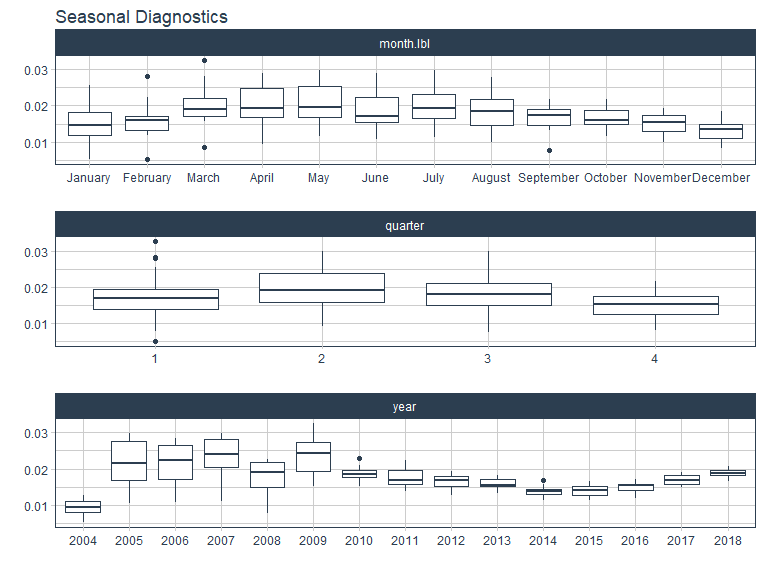


**Fig. S17.** Tuberculosis mortality rates by month, quarter, and year for 31 provinces in mainland China.
